# Spectroscopic Methods in Evaluation of Antioxidant Potential, Enzyme Inhibition, Cytotoxicity, and Antimicrobial Activity of the Synthesized *N*^3^-Substituted Amidrazones

**DOI:** 10.3390/ijms27020746

**Published:** 2026-01-12

**Authors:** Renata Paprocka, Leszek Pazderski, Jolanta Kutkowska, Iqra Naeem, Amna Shahid Awan, Zahid Mushtaq, Aleksandra Szydłowska-Czerniak

**Affiliations:** 1 Department of Organic Chemistry, Faculty of Pharmacy, Collegium Medicum in Bydgoszcz, Nicolaus Copernicus University in Toruń, Jurasza Str. 2, 85-089 Bydgoszcz, Poland; renata.bursa@cm.umk.pl; 2 Department of Analytical Chemistry and Applied Spectroscopy, Faculty of Chemistry, Nicolaus Copernicus University in Toruń, Gagarina Str. 7, 87-100 Toruń, Poland; leszekp@chem.umk.pl; 3 Department of Genetics and Microbiology, Institute of Biological Sciences, Maria Curie-Skłodowska University, Akademicka Str. 19, 20-033 Lublin, Poland; jolanta.kutkowska@mail.umcs.pl; 4 Bioactive Molecules Research Lab (BMRL), Department of Biochemistry, University of Agriculture Faisalabad, Faisalabad 38000, Pakistan; iqranaeem305@gmail.com (I.N.); awanamnashahid@gmail.com (A.S.A.); zahidmushtaquaf@uaf.edu.pk (Z.M.)

**Keywords:** carbohydrazonamides, synthesis, antioxidant activity, enzyme inhibitors, bactericidal and fungicidal properties, NMR spectroscopy, spectrophotometry, chemometric analysis

## Abstract

Seven amidrazones containing a characteristic NH_2_–N=C(Ar^1^)–NHAr^2^ moiety, where Ar^1^, Ar^2^ are phenyl, 4-methylphenyl, 4-nitrophenyl, 2-pyridyl, and 4-pyridyl substituents, denoted as **2a**–**2g**, were synthesized by the reactions between thioamides and hydrazine. Their molecular structures were confirmed by ^1^H, ^13^C, ^1^H-^13^C HMQC, ^1^H-^13^C HMBC, and ^1^H-^15^N HMBC NMR spectroscopy, with complete assignment of the detected signals, as well as by high-resolution mass spectra. The biological activity of all compounds was studied, exhibiting antioxidant properties determined by 2,2-diphenyl-1-picrylhydrazyl (DPPH) and ferric reducing antioxidant power (FRAP) methods, inhibitory potential against digestive tract enzymes (α-amylase, lipase, pepsin), cytotoxicity (hemolysis), and antimicrobial activities (against Gram-positive and Gram-negative bacteria, and a fungus). The antioxidant activity of the studied amidrazones varied from 83.34% to 93.27% and 1.01–5.79 mM FeSO_4_ for the DPPH and FRAP methods, respectively. Moreover, these derivatives revealed inhibition potential against α-amylase (28.6–86.8%), lipase (28.0–60.0%), and pepsin (34.1–76.6%), which increased when increasing their concentrations from 0.2 to 1 mg/mL. Among them, compound **2d** (possessing 2-pyridyl and 4-nitrophenyl substituents) stood out in particular, as a potent antioxidant (DPPH = 90.43%, FRAP = 4.73 Mm FeSO_4_) with the highest activity against Gram-positive bacteria: *S. aureus* (MIC = 64 μg/mL), *G. rubripertincta* (MIC = 64 μg/mL), and fungus: *C. albicans* (MIC = 32 μg/mL); high α-amylase (86.8%) inhibition at the highest concentration (1 mg/mL); and lipase (38.0%) and pepsin (43.8%) inhibition at the lowest concentration (0.2 mg/mL). The obtained results were analyzed by unsupervised multivariate techniques to confirm significant differences in the biological activity of amidrazones depending on the Ar^1^ and Ar^2^ substituents.

## 1. Introduction

**Figure 1:**
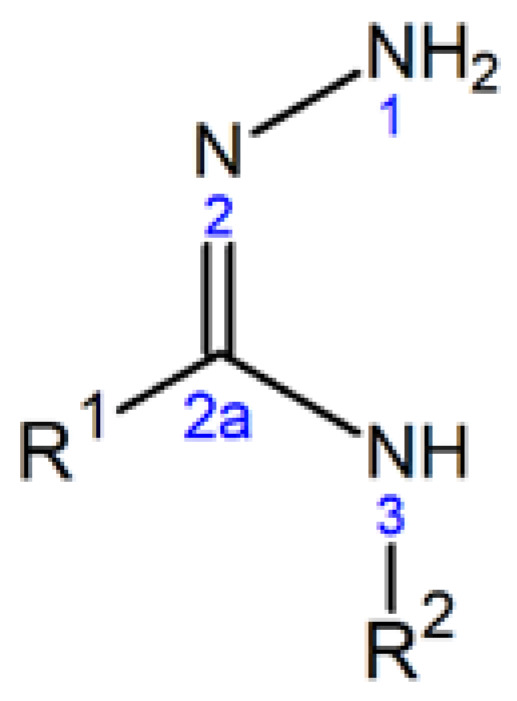
Amidrazones, their numbering scheme, and R^1^, R^2^ substituents.

Amidrazones containing a characteristic N–N=C–N moiety, whose general molecular formula (together with the numbering scheme and the optional R^1^, R^2^ substituents) is presented in Figure 1, are useful precursors in the synthesis of various five-, six-, and seven-membered heterocycles [1].

Over the past few decades, numerous studies have focused on the synthesis of amidrazones, their derivatives and metal complexes (all in the broadest possible meaning, i.e., involving organics, organometallics, coordination compounds, etc.), as well as on the determination of their biological properties, including antitumor, antimicrobial, antimalarial, antiviral, anti-inflammatory, analgesic, anticonvulsant and antinociceptive activities [2,3,4,5,6,7,8,9,10,11,12,13,14,15,16].

**Figure 2:**
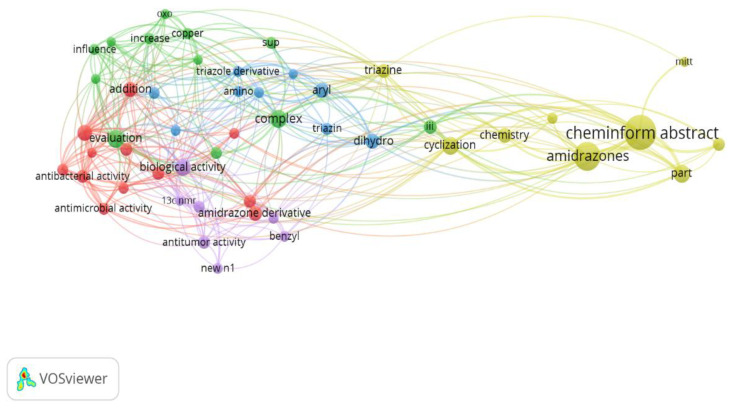
The co-occurrence map of terms indicating the publication trends on amidrazones. Different colors represent terms belonging to different clusters. Connecting lines indicate the strongest co-occurrence links between terms.

In order to identify the present trends in scientific interest on the above class of chemicals, we have generated, using VOSViewer (version 1.6.20) software, a two-dimensional co-occurrence map, based on the frequency of the term “amidrazones”, with the examination of the 2825 terms, with 81 meeting the threshold of appearing at least five times (Figure 2).

This analysis revealed five distinct clusters, highlighting trends in the research of these compounds, including synthesis and structural studies, methods of characterization, and biological activities (antibacterial, antimicrobial, and antitumor properties).

In particular, some authors have argued that various amidrazone derivatives and metal complexes exhibit significant antitumor properties against several cancer cell lines, including those of breast, colon, lung, and prostate cancers, as well as leukemia and epidermal carcinoma [9,10,11].

Furthermore, antimicrobial properties of this group of compounds were also intensively studied. Thus, 1*H*-pyrrole-2,5-dione derivatives, containing *N*-bonded amidrazone substituents, were found to be active against *Enterococcus faecalis*, *Escherichia coli*, *Micrococcus luteus*, *Mycobacterium smegmatis*, *Nocardia corralina*, *Pseudomonas aeruginosa*, *Staphylococcus aureus*, and *Yersinia enterocolitica* bacteria [13], whereas amidrazone amides of cyclohex-1-ene-1-carboxylic acid were active against the bacterial strains of *E. coli*, *Klebsiella pneumoniae*, *M. smegmatis*, *S. aureus* and *Y. enterocolitica*, as well as the fungal strain of *Candida albicans* [16]. Moreover, *N*^1^-2,4-dihydroxybenzenecarbothio-*N*^3^-phenylacylamidrazones exhibited antifungal action against various dermatophytes, yeasts, and molds [4], while 1-(piperidin-1-yl)-*N*^2^-arylamidrazones exhibited both antibacterial (against *Bacillus subtilis*, *E. coli*, *P. aeruginosa*, and *S. aureus*) and antifungal (against *Aspergillus fumigatus* and *C. albicans*) properties [7].

Then, an amidrazone-derived Au(III) complex demonstrated antibacterial activity against *B. subtilis* and *S. aureus*, as well as against a drug-resistant strain of *K. pneumoniae* [12]. In contrast, another amidrazone derivative and its Cu(II) complex had only low antibacterial activity, being also devoid of antifungal activity [9]. Finally, the antibacterial properties of 85 amidrazones and various amidrazone-derived compounds against some Gram-positive bacteria (*E. faecalis*, *M. luteus*, *M. smegmatis*, *N. corallina,* and *S. aureus*) were predicted using generalized linear models [14].

In addition to this, the above-mentioned amidrazone-derived 1*H*-pyrrole-2,5-diones and cyclohex-1-ene-1-carboxylic amides also possessed anti-inflammatory properties, related to the reduction in human peripheral-blood mononuclear cell (PBMC) proliferation, as well as to the inhibition of tumor necrosis factor TNF-α and IL-6, IL-10, and IL-1β pro-inflammatory cytokines production [13,16].

The illustration of the biological activity of amidrazones may be the fact that their best-known representative, called delpazolid (which contains the N–N=C–N moiety inside the alicyclic ring, which is different from open aliphatic chain molecules studied upon the present study) [17], has a pharmacological action currently being studied in clinical trials in combination therapy for the treatment of pulmonary tuberculosis [18]. Furthermore, other cyclic amidrazones are becoming increasingly popular in the design of new potential drugs [19,20].

**Scheme 1:**
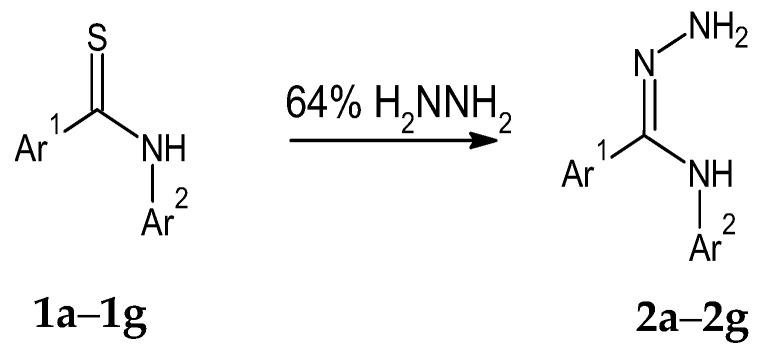
Synthesis of amidrazones **2a**–**2g** from thioamides **1a**–**1g** and hydrazine.

In the present study, we have focused on the biological activity of seven amidrazones containing, as R^1^ and R^2^ substituents (Figure 1), various aryl or heteroaryl rings (in the further part of the work marked as Ar^1^ and Ar^2^): phenyl, 4-methylphenyl, 4-nitrophenyl, 2-pyridyl, and 4-pyridyl. These compounds are herewith denoted as **2a**–**2g**, following the combination of Ar^1^ and Ar^2^ as shown in Table 1 (the symbols **1a**–**1g** are reserved for the corresponding thioamides, from which the amidrazones were synthesized as shown in Scheme 1.

It is a kind of paradox that, although **2a**–**2g** compounds were used by many authors [21,22,23,24,25,26,27,28,29,30,31,32,33] as starting points for the preparation of some other, more advanced organic species often exhibiting their own biological activity, the concerned amidrazones themselves were not investigated from this viewpoint at all.

It is also important that, despite the fact that many amidrazones and their derivatives were already studied with respect to their antitumor, antimicrobial, antimalarial, antiviral, anti-inflammatory, analgesic, anticonvulsant and antinociceptive properties (as we have already explained), to the best of our knowledge there is no literature reference on the determination of their antioxidant activity or digestive tract enzymes inhibition.

Furthermore, some of the presently studied amidrazones (namely **2c**–**2e** and **2g**) still do not have any synthesis characteristics at all, e.g., in the Reaxys database [34]—and this lack also concerns basic information, such as their melting point.

Finally, the spectroscopic characterization of amidrazones **2a**–**2g** is insufficient. For example, despite the relative simplicity of their molecules, these nitrogen-containing compounds have never been studied by ^15^N NMR; consequently, their ^15^N chemical shifts are unavailable.

Therefore, the main goal of the present work was the study of the biological activity of amidrazones **2a**–**2g** that included the determination of the following: (1) antioxidant properties using two analytical assays (DPPH—2,2-diphenyl-1-picrylhydrazyl and FRAP—ferric-reducing antioxidant power); (2) inhibitory activity for three digestive tract enzymes: α-amylase, lipase, and pepsin; (3) cytotoxic activity; and (4) antibacterial and antifungal activity. Then, this wide study was followed by the chemometric multivariate analyses (PCA—principal component analysis and HCA—hierarchical cluster analysis). Moreover, Pearson correlations were calculated, and the color correlation matrix was generated to analyze the relationships among the evaluated biological properties of the synthesized compounds. Additionally, the ^1^H-^15^N HMBC-NMR spectra of **2a**–**2g** were measured, yielding the ^15^N chemical shifts for the observable nitrogen atoms.

## 2. Results and Discussion

### 2.1. The Synthesis of Amidrazones ***2a***–***2g***

Amidrazones **2a**–**2g** were obtained by the modified Spassov method [35] in the reactions of thioamides **1a**–**1g** with 64% hydrazine hydrate solution, as shown in Scheme 1.

The modification of the synthesis involved the usage of a lower concentration of hydrazine hydrate (64% instead of 80%); thus, similar product yields were obtained upon the reduction in the reagents’ toxicity and costs.

### 2.2. NMR Study of Amidrazones ***2a***–***2g***

Amidrazones **2a**–**2g** were characterized by ^1^H, ^13^C, ^1^H-^13^C HMQC, ^1^H-^13^C HMBC, and ^1^H-^15^N HMBC NMR spectra (in DMSO-d6), with the assignment of all signals. The ^1^H, ^13^C, and ^15^N chemical shifts are listed in Table 2, Table 3 and Table 4; the positions inside the Ar^1^ and Ar^2^ substituents are numbered as 1′…6′ and 1″…6″, respectively.

In the past, we published the assigned ^1^H and ^13^C NMR chemical shifts (but not the ^15^N ones) for amidrazones **2a**–**2f** (in the Supplementary Data of our previous paper [13]: https://www.mdpi.com/article/10.3390/molecules27092891/s1 (accessed on 4 January 2026)), whereas those for **2g**, determined in this work, are new. The one-dimensional ^1^H and ^13^C NMR spectra, as well as two-dimensional ^1^H-^13^C HMQC and ^1^H-^13^C HMBC spectra for **2g**, together with the ^1^H-^15^N HMBC ones for all **2a**–**2g** species, are presented in the Supplementary Data (Part A—Figures S1–S5, and Part B—Figures S6–S12).

The ^1^H and ^13^C NMR signals for **2g** were assigned in the same way as for **2a**–**2f** [13], i.e., by ^1^H-^13^C HMQC (one-bond correlations) and ^1^H-^13^C HMBC (long-range correlations) methods. The ^13^C chemical shifts of **2g** follow an analogous pattern as in the previously studied amidrazones: the values for C1′–C3′ are nearly identical to those in **2f** (the same Ar^1^ = 4-pyridyl substituent), while those for C1″–C4″ are similar to those in **2c** (the same Ar^2^ = 4-methylphenyl substituent). Then, the ^13^C chemical shifts for C2a in **2g** and **2a**–**2f** are in the relatively narrow ca. 135–139 ppm range, their similarity reflecting the presence of the same >C= moiety.

The ^15^N signals were assigned based on the correlations in the ^1^H-^15^N HMBC spectra. They were usually observed at the distance of two (N2′H3′, N4′H3′, N2″H3″) and/or three (N2′H4′, N3H2″, NO_2_-H3″) bonds. For example, in the ^1^H-^15^N HMBC spectrum of the new **2g** compound, the H3′ atom correlates with N4′ (two bonds distance: H3′-C3′-N4′), while the H2″ one correlates with N3 (three bonds distance: H2″-C2″-C1″-N3); moreover, from the viewpoint of both concerned hydrogens these are the only possible correlations (for any others, the distance to the nearest nitrogen(s) would exceed four bonds)—which unambiguously indicates N4′ and N3, respectively. Additionally, for **2b** and **2e** where, in contrast to **2a**, **2c**–**2d**, and **2f**–**2g**, the H1 and H3 signals were much less broadened (indicating the lack of mobility of NH_2_ and NH protons, at least in the NMR time scale), the interactions over one bond were also observed (N1H1, i.e., inside the NH_2_ group; and N3H3, i.e., inside the NH group). Finally, the unprotonated N2 atom was never detected, most likely because of the too-long distance to the nearest protons in Ar^1^ and Ar^2^ substituents (four bonds to H2′ and five bonds to H2″).

The N3 signal in all **2a**–**2g** amidrazones appears in the ca. −302 to ca. −285 ppm range. These values are comparable to the ^15^N chemical shift for *N*-methylaniline (containing the analogous Ph-NH-C moiety), which is 53.0 ppm (also in DMSO-d_6_) as referenced to liquid ammonia, i.e., −328.7 ppm in respect to neat nitromethane [36]; the relative shift of −381.7 ppm between liquid NH_3_ and neat CH_3_NO_2_ was taken into account [37].

The N3 chemical shift (δ^N3^) is sensitive to the changes in Ar^1^ and/or Ar^2^ substituents. It can be especially exemplified by **2b**–**2e** containing the same Ar^1^ = 2-pyridyl and different Ar^2^ substituents: the Ar^2^ = phenyl → Ar^2^ = 4-methylphenyl (**2b** → **2c**) replacement results in a ca. 3 ppm decrease in δ^N3^ (−296.3 ppm → −299.3 ppm), whereas the Ar^2^ = phenyl → Ar^2^ = 4-nitrophenyl or 2-pyridyl (**2b** → **2d** or **2e**) transition results in a ca. 9–11 ppm increase in δ^N3^ (−296.3 ppm → −287.0 ppm or −285.0 ppm). This is understandable, as the former ^15^N shielding effect corresponds to the introduction of the electron-donating methyl group (4-CH_3_), while the latter ^15^N, a deshielding one, corresponds to the presence of the electron-withdrawing nitro group (4-NO_2_) or a heterocyclic nitrogen atom (in the 2-pyridyl moiety). A similar, although noticeably weaker N3 shielding phenomenon (δ^N3^ decrease by 0.5 ppm: −301.2 ppm → −301.7 ppm) was related to the introduction of the electron-donating 4-CH_3_ side group into Ar^2^ (Ar^2^ = phenyl → Ar^2^ = 4-methylphenyl) upon the **2f** → **2g** replacement, i.e., when Ar^1^ = 4-pyridyl remained identical.

In contrast, the δ^N3^ changes following variations inside Ar^1^ (Ar^1^ = phenyl → Ar^2^ = 2-pyridyl or 4-pyridyl), upon the same Ar^2^ = phenyl (thus, **2a** → **2b** or **2f**), were less characteristic (−296.7 ppm → −296.3 ppm or −301.2 ppm, respectively), although both 2-pyridyl and 4-pyridyl substituents are electron-withdrawing. Most likely, the structural changes concerning Ar^1^ (bound to C2a) are simply less important from the viewpoint of the N3 atom than those occurring within Ar^2^ (bound just to N3); thus, the resulting shielding/deshielding effects at this nitrogen are of various signs and small absolute magnitude.

Then, for **2b**–**2e** and **2f**–**2g**, the N2′ or N4′ atoms (in Ar^1^ = 2-pyridyl or 4-pyridyl) are present in the respective ranges from ca. −77 to ca. −74 ppm or from ca. −70 to ca. −69 ppm. These δ^N2′^ and δ^N4′^ values are generally similar to those for 2-methylpyridine and 4-methylpyridine (containing the same 2-Py-C or 4-Py-C moiety), which are 315.7 ppm and 303.4 ppm (both in DMSO-d_6_), respectively, as referenced to liquid ammonia, i.e., −66.0 ppm and −78.3 ppm in respect to neat nitromethane [36,37]. However, for these Ar^1^ substituents, the 2-pyridyl nitrogens are by ca. 4–8 ppm more shielded than the 4-pyridyl ones; as the best examples may serve the pairs of **2b** and **2f** (with the same Ar^2^ = phenyl): δ^N2′^ = −74.3 ppm vs. δ^N4′^ = −69.4 ppm, as well as **2c** and **2g** (with the same Ar^2^ = 4-methylphenyl): δ^N2′^ = −76.9 ppm vs. δ^N4′^ = −69.1 ppm. It is interesting that in the case of the presently studied amidrazones, such a relationship is opposite to that reported for simple methylpyridines (δ^N2′^ = −66.0 ppm in 2-methylpyridine vs. δ^N4′^ = −78.3 ppm in 4-methylpyridine); this is surprising but certain.

Finally, as it could be expected, the Ar^2^ changes (phenyl → 4-methylphenyl → 4-nitrophenyl → 2-pyridyl and phenyl → 4-methylphenyl, in the corresponding series of **2b** → **2c** → **2d** → **2e** and **2f** → **2g**) have rather negligible impact at the δ^N2′^ and δ^N4′^ chemical shifts (−74.3 ppm → −76.9 ppm → −74.4 ppm → −76.8 ppm and −69.4 ppm → −69.1 ppm, respectively), most likely owing to the large distance between N2′ and N4′ atoms from the N3-bonded Ar^2^ ring system.

In contrast, for **2e** (the only species with 2-pyridyl as Ar^2^), the N2″ signal occurs at −108.9 ppm, which is ca. 30–35 ppm smaller than those of N2′ for **2b**–**2e** (all with 2-pyridyl as Ar^1^); it is also ca. 43 ppm less than for 2-methylpyridine (δ^N2′^ = −66.0 ppm). Such a strong deshielding of N2″ compared to N2′ in **2e** (−108.9 ppm vs. −76.8 ppm) is probably caused by linking of the electron-acceptor -NH- group to Ar^2^, while Ar^1^ is carbon-bonded.

Finally, for **2d**, the -NO_2_ side group (in Ar^2^ = 4-nitrophenyl) occurs at −8.8 ppm. This parameter is very close to that for nitrobenzene: −9.8 ppm, for 4-nitroaniline (i.e., 1-amino-4-nitrobenzene): −9.6 ppm, and for *N*,*N*-dimethyl-4-nitroaniline (i.e., 1-dimethylamino-4-nitrobenzene): −10.4 ppm (all in DMSO-d_6_, the ^15^N chemical shifts referenced directly to neat nitromethane) [38,39,40].

### 2.3. Biological Activity of Amidrazones ***2a***–***2g***

#### 2.3.1. Antioxidant Activity of Amidrazones **2a**–**2g**

**Figure 3:**
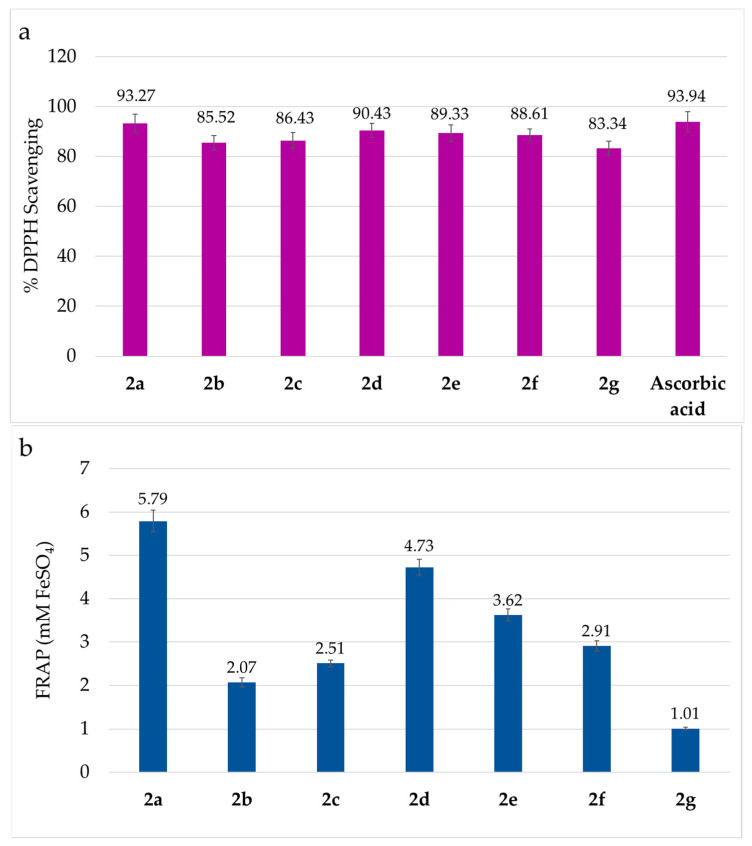
Antioxidant activity of amidrazones **2a**–**2g** determined by (**a**) DPPH assay (at amidrazones concentration 1 mg/mL) and (**b**) FRAP assay (at amidrazones concentration 4 mM). Results are expressed as mean ± SEM of three independent experiments.

The antioxidant activity of compounds **2a**–**2g** was determined using two spectrophotometric methods: the DPPH radical scavenging assay, which measures the ability of amidrazones to scavenge DPPH radicals, causing a discoloration of the DPPH solution; and the FRAP assay, which measures the reducing ability of these compounds by converting the colorless Fe^3+^–TPTZ (2,4,6-tripyridyl-s-triazine) complex into the intensely blue Fe^2+^–TPTZ complex. The obtained DPPH and FRAP results are presented in Figure 3a,b.

All amidrazones **2a**–**2g** showed high antioxidant potential, with the ability to inhibit free radicals from 83.34% to 93.27% in the DPPH assay (Figure 3a), while the FRAP values ranged between 1.01 and 5.79 mM FeSO_4_ (Figure 3b).

The highest DPPH activity was exhibited by **2a** (93.27%) and **2d** (90.43%), being comparable to the reference ascorbic acid (93.94%). Interestingly, the same amidrazones demonstrated the highest FRAP results: 5.79 mM FeSO_4_ for **2a** and 4.73 mM FeSO_4_ for **2d**.

Generally, the order of antioxidant activity of compounds **2a**–**2g** determined by the DPPH and FRAP methods is the same: **2a** > **2d** > **2e** > **2f** > **2c** > **2b** > **2g**; therefore, the DPPH and FRAP results are consistent. Based on this, the influence of individual substituents present in amidrazone derivatives on their antioxidant properties can be observed. As already mentioned, a compound **2a**, containing two phenyl rings, weak electron-donating substituents at Ar^1^ and Ar^2^ positions, had the highest antioxidant properties. However, the replacement of the phenyl group by 2-pyridyl (**2a** → **2b**–**2e**) or 4-pyridyl (**2a** → **2f**–**2g**) substituent at the Ar^1^ position decreased the antioxidant activity of amidrazones **2b**–**2g** significantly (this is particularly visible in the FRAP results). This indicates that electron-withdrawing substituents at the Ar^1^ position reduce the antioxidant activity of the tested compounds. Meanwhile, for Ar^2^ position (when to compare amidrazones having the same Ar^1^) the replacement of phenyl group by electron-withdrawing substituents, such as 4-nitrophenyl (**2b** → **2d**) or 2-pyridyl (**2b** → **2e**), enhanced antioxidant activity (both examples upon Ar^1^ = 2-pyridyl), whereas the impact of analogous phenyl → 4-methylphenyl (the electron-donating group) transition is more variable, including either enhancement (**2b** → **2c**, upon Ar^1^ = 2-pyridyl) or weakening (**2f** → **2g**, upon Ar^1^ = 4-pyridyl) of antioxidant properties.

The DPPH and/or FRAP methods have already been used to determine the antioxidant properties of many organic compounds, including nitrogen-containing species, such as vanillin-derived amines [41] and sinaptic acid anilides [42]. It confirms their usefulness in the research on the biological activity of compounds of natural and synthetic origin. However, as mentioned, the DPPH and FRAP methods have never been applied to analogous studies of amidrazones.

#### 2.3.2. Inhibitory Activity of Amidrazones **2a**–**2g** Against Enzymes

**Figure 4:**
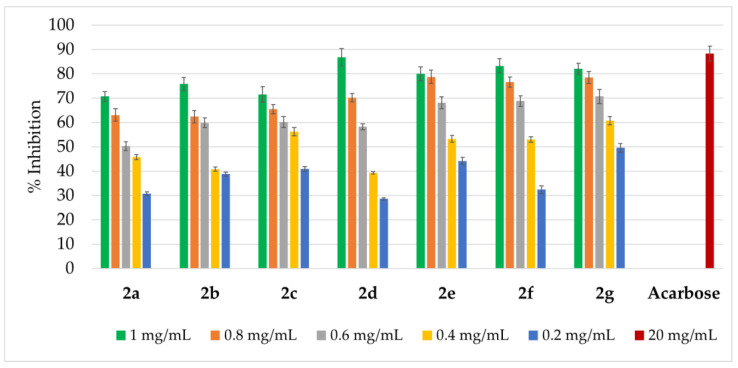
α-amylase-inhibition activity of amidrazones **2a**–**2g**. Results are expressed as mean ± SEM of three independent experiments.

**Figure 5:**
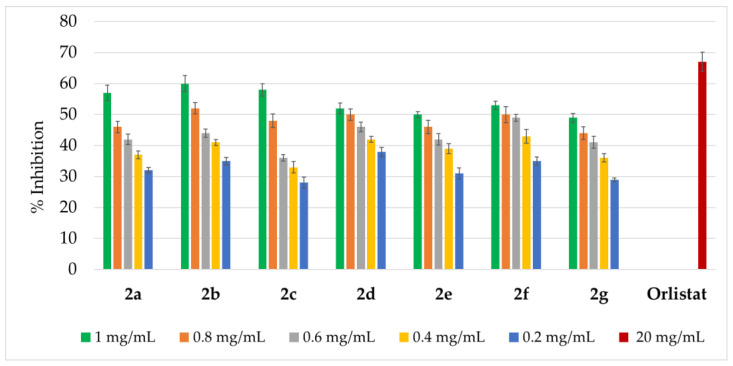
Lipase-inhibition activity of amidrazones **2a**–**2g**. Results are expressed as mean ± SEM of three independent experiments.

**Figure 6:**
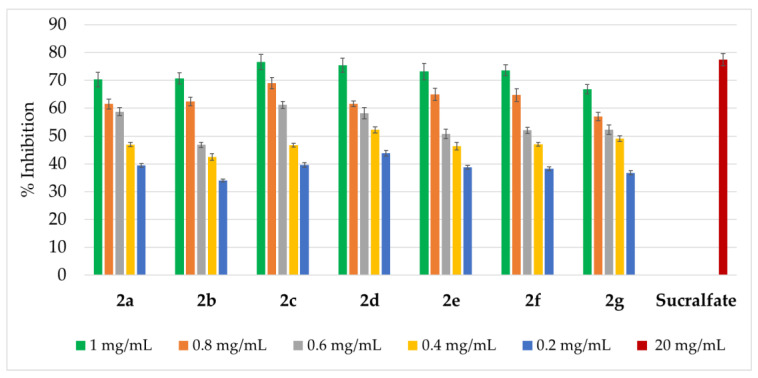
Pepsin-inhibition activity of amidrazones **2a**–**2g**. Results are expressed as mean ± SEM of three independent experiments.

The inhibitory activity of compounds **2a**–**2g** was tested against three digestive tract enzymes: α-amylase, lipase, and pepsin, and the obtained results are presented in Figure 4, Figure 5 and Figure 6.

Amidrazones **2a**–**2g** exhibited α-amylase inhibition in a range of approximately 28.6–86.8%, with activity increasing with concentration (Figure 4).

In the case of the highest amidrazones concentration (1 mg/mL), the α-amylase inhibition rate was within a 70.7–86.8% range, being the strongest for **2d**: 86.8% (which is similar to a reference drug acarbose [43] at the 20 mg/mL dose: 88.3%). It is worth noting that all compounds at a concentration of 1 mg/mL showed an inhibition rate above 70%. However, derivatives **2e**–**2g** exhibited relatively high inhibiting activity also at medium concentrations of 0.6–0.8 mg/mL (e.g., >75% at 0.8 mg/mL). This suggests that substituents, such as a 4-pyridine ring in the Ar^1^ position (**2f**–**2g**) or two 2-pyridine rings (**2e**), may also have a beneficial effect on α-amylase inhibitory activity.

Moreover, the synthesized amidrazones **2a**–**2g** inhibited lipase activity by 28.0–60.0% (Figure 5).

It is noteworthy that the lipase inhibition rate was in the 49.0–60.0% range for the highest concentration (1 mg/mL) of the synthesized compounds, being the strongest for **2a**–**2c**: 57.0–60.0% (which is ca. 0.1 times lower than for a reference drug orlistat [44] at the 20 mg/mL dose: 67.0%).

Finally, compounds **2a**–**2g** exhibited pepsin inhibition in a range of approximately 34.1–76.6%, with activity increasing with concentration (Figure 6).

As can be seen, for the highest concentration (1 mg/mL) of amidrazones, the pepsin inhibition rate was in the 66.9–76.6% range, being the strongest for **2c**: 76.6% (which is close to a reference drug, sucralfate [45] at the 20 mg/mL dose: 77.4%). However, all compounds at a concentration of 1 mg/mL, except **2g**, with 4-pyridyl and 4-methylphenyl as Ar^1^ and Ar^2^ substituents, showed an inhibition rate above 70%.

In summary, all amidrazones **2a**–**2g** revealed dose-dependent inhibitory effects on the three gastrointestinal enzymes: α-amylase, lipase, and pepsin. Some of them, applied at a dose of 1 mg/mL, exhibited inhibition activities comparable to those of the reference drugs acarbose, orlistat, and sucralfate (used at a twenty-fold higher dose of 20 mg/mL). Among all the synthesized amidrazones, compound **2d** containing 2-pyridyl and 4-nitrophenyl in Ar^1^ and Ar^2^ positions, respectively, stood out in particular, with the highest α-amylase inhibition at the highest concentration (1 mg/mL) and lipase and pepsin inhibition at the lowest concentration (0.2 mg/mL).

To the best of our knowledge, no reports on the impact of amidrazones on lipase, pepsin, and amylase inhibitory activity have been published to date. However, analogous effects were reported for a few different enzymes, but in the case of some other amidrazones (not those of **2a**–**2g**) and also their derivatives. In particular, some amidrazones were capable of inactivating soybean lipoxygenase-1 [46]. Moreover, several compounds containing the amidrazone moiety were described as acetylcholinesterase (AChE) or butyrylocholinesterase (BChE) inhibitors (potentially valuable for Alzheimer’s disease treatment) [47,48], while a few others showed the ability to inhibit furins, trypsin, and thrombin [49,50].

Thus, the present data, combined with the reports of previous authors, indicate the possibility of using amidrazones and their derivatives to inhibit various enzymatic processes.

#### 2.3.3. Cytotoxic Activity of Amidrazones **2a**–**2g**

To determine the cytotoxicity of amidrazones **2a**–**2g**, a hemolysis test was performed. Hemolysis is the rupture of red blood cells (erythrocytes), indicating cytotoxic effects on these cells [51]. Triton X-100 [52] (1 mg/mL), used as a reference, caused 91.6% hemolysis.

**Figure 7:**
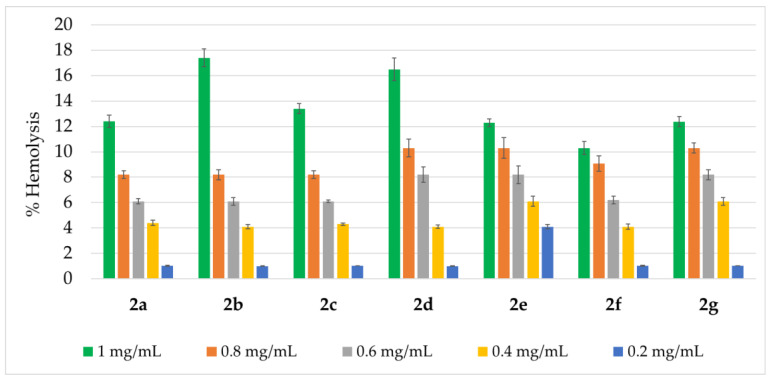
Cytotoxic activity of amidrazones **2a**–**2g** expressed as percentage of hemolysis. Results are expressed as mean ± SEM of three independent experiments.

The percentage of hemolysis of amidrazones **2a**–**2g**, depending on the concentration used, is illustrated in Figure 7.

The most potent cytotoxic activity was observed for two amidrazones at the highest concentration (1 mg/mL), i.e., **2b** and **2d** (17.4% and 16.5%, respectively), which featured a 2-pyridyl group in the Ar^1^ position and phenyl and 4-nitrophenyl groups, respectively, in the Ar^2^ position. However, at this concentration, other investigated amidrazones were able to rupture erythrocytes, with a range of 10.3% to 13.4%. Importantly, all amidrazones at the lower concentrations of 0.2–0.4 mg/mL exhibited low cytotoxicity (1.0–6.1%).

To the best of our knowledge, the hemolytic activity of amidrazones has not been previously studied. Among compounds containing a hydrazine moiety (which is a part of the amidrazone system), phenylhydrazine showed several times higher hemolytic activity (ca. 40.5%), at ca. 0.6–0.7 mg/mL concentrations [53].

#### 2.3.4. Antibacterial and Antifungal Activity of Amidrazones **2a**–**2g**

The disk diffusion method was used to qualitatively test the antimicrobial activity of seven amidrazone derivatives **2a**–**2g** against five Gram-positive bacterial strains (*S. aureus*, *M. luteus*, *E. faecalis*, *M. smegmatis*, and *G. rubripertincta*) and four Gram-negative bacterial strains (*E. coli*, *Y. enterocolitica*, *K. pneumoniae*, and *P. aeruginosa*), as well as a *C. albicans* fungal strain. To determine the minimum inhibitory concentration (MIC) of each synthesized compound, the broth microdilution method was employed. The results of the antimicrobial activity test for the amidrazone derivatives are presented in Table 5.

The strongest antibacterial activity against all Gram-positive bacterial strains was exhibited by **2d**, which features 2-pyridyl and 4-nitrophenyl groups in the Ar^1^ and Ar^2^ positions, respectively. The MIC values were 64 µg/mL for *S. aureus* and *G. rubripertincta*, as well as 128 mg/mL for *M. luteus*, *E. faecalis*, and *M. smegmatis*. However, the same amidrazone **2d** was less effective against Gram-negative bacterial strains, and, unfortunately, showed no inhibitory effect on the Gram-negative bacterium *P. aeruginosa* (MIC > 512 µg/mL). On the contrary, the compound **2d** revealed the best antifungal activity with an MIC parameter against *C. albicans* of 32 µg/mL, i.e., 4 to 8 times less than for all other compounds (MIC = 128–256 µg/mL).

The least favorable substituent arrangement with respect to antimicrobial activity was the presence of two 2-pyridyl rings (**2e**) or the 2-pyridyl and phenyl ones (**2b**), their MIC values being at least 256 µg/mL. Then, the antimicrobial activity of **2b** and **2d** (both with the same Ar^1^ = 2-pyridyl substituent) was significantly enhanced upon the introduction of the 4-NO_2_ side group into the Ar^2^ phenyl ring, as indicated by the respective MIC decreases (256 or 512 µg/mL → 64 µg/mL for *S. aureus* and *G. rubripertincta*; 256 µg/mL → 32 µg/mL for *C. albicans*).

In our previous and the most recent papers, we described the antibacterial properties of some acyl, pyrrole-2,5-dione, and 1,2,4-triazole derivatives, obtained in the reactions of amidrazones **2a**–**2g** with cyclic anhydrides. These compounds exhibited variable activity (in general, their MIC values were in the 64–512 µg/mL range), being usually more active against Gram-positive than Gram-negative bacterial strains [13,16,33,54].

Then, we also reported the antifungal properties of some acyl derivatives, formed in the reactions of amidrazones **2a**–**2g** with maleic anhydride [54] or 3,4,5,6-tetrahydrophthalic anhydride [16], their MIC values being 256–512 mg/mL against *C. albicans*. Thus, all the presently studied **2a**–**2g** compounds exhibit significantly higher antifungal activity, which indicates a larger potential of amidrazones possessing the unsubstituted hydrazine group.

**Figure 8:**
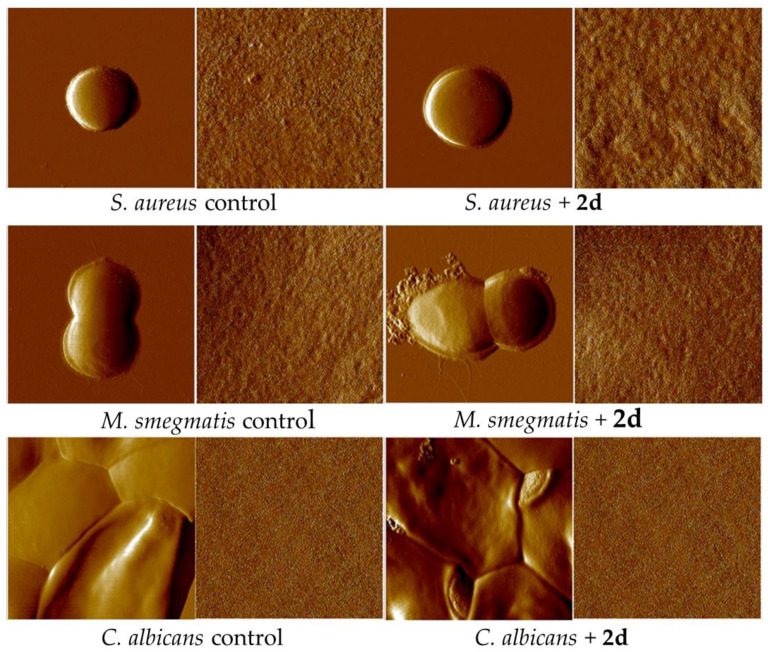
AFM images of control and amidrazone **2d**-treated *S. aureus*, *M. smegmatis*, and *C. albicans* cells.

Additionally, atomic force microscopy (AFM) was used to visualize the effect of the most potent amidrazone **2d** on the cell walls of representative bacterial strains, such as *S. aureus* and *M. smegmatis*, as well as the yeast strain *C. albicans* (Figure 8). This method is particularly useful because it allows for imaging and measurement of the mechanical properties of living cells under physiological conditions [55].

In the present case, bacterial and fungal strains were grown in the presence of **2d** at a concentration twice lower than the respective MIC value. The AFM analysis revealed that treatment with this amidrazone for 12 h induced some morphological changes in the bacterial walls of *M. smegmatis* and *C. albicans*, resulting in an increased surface roughness of the cells (1.25-fold and 1.14-fold, respectively). The surface roughness of the cell was calculated using the root mean square (RMS) value, which represents the standard deviation of the height distribution within a 1 × 1 μm^2^ image area. The increase in the numerical surface roughness suggests the efficiency of **2d** in interacting with the cellular membrane.

In particular, the exposure of *C. albicans* cells to **2d** led to slight irregularities on the cell surface and the release of small vesicles, whereas the surface of the control cells, having a smooth surface regular shape, was covered with small, uniform grains.

Then, the surface of *M. smegmatis* cells treated with **2d** was less smooth than that of the control cells (the culture without the addition of **2d**); numerous irregularities and protrusions were noticeable.

Finally, **2d** only slightly affected the shape of *S. aureus* cells and the roughness of their surface (RMS = 1.02), which was relatively smooth and contained no pores or cracks, thus being almost indistinguishable from the control cells.

### 2.4. Chemometric Analysis

Principal component analysis (PCA) and hierarchical cluster analysis (HCA), as representatives of unsupervised methods, were employed to assess similarities/dissimilarities between seven amidrazones **2a**–**2g** differing in the Ar^1^ and Ar^2^ substituents, as well as to project them in a two-dimensional factor plane based on their biological activities including antioxidant, enzymes inhibition, cytotoxic, and antimicrobial (antibacterial and antifungal) properties.

#### 2.4.1. Principal Component Analysis

**Figure 9:**
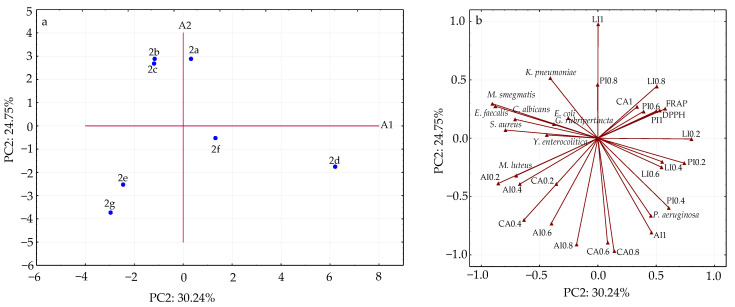
Score plot (**a**) and loading plot (**b**) of the first two principal components (PC1 and PC2) for the classification of seven amidrazones **2a**–**2g**, and describing the variation among their biological properties.

The PCA was utilized to compare seven amidrazones **2a**–**2g** based on their biological parameters, and to find the relationships between all investigated biological descriptors (antioxidant activity determined by DPPH and FRAP methods, α-amylase, lipase, and pepsin inhibition, cytotoxic activity, and antibacterial and antifungal activity). The maximum number of principal components (PCs) was set at six of the thirty-two PCs, which gave eigenvalues greater than 1.00 (9.68, 7.92, 5.44, 4.28, 2.50, 2.19). However, the first two components contributed 54.99% of the total variation for the thirty-two characters under study. Therefore, only the first two PCs were used to understand the similarities or dissimilarities of the seven amidrazones **2a**–**2g** as illustrated in the score plot in Figure 9a. Consequently, the relationships between the first two principal components and the studied variables were presented graphically by the loading plot in Figure 9b.

The PC1 was positively correlated with antioxidant activity determined by DPPH (0.5315) or FRAP (0.5788) methods, and with lipase and pepsin inhibitory activity (0.5033–0.8011 and 0.3918–0.7423, respectively) at amidrazone concentrations (0.2–0.8 mg/mL), while negatively correlated with antimicrobial activity against the four Gram-positive bacterial strains (*S. aureus*, *M. luteus*, *E. faecalis*, *M. smegmatis*), fungal strain (*C. albicans*) (−0.6999 to −0.9073), and with α-amylase inhibition (−0.6672 and −0.8521) at low amidrazone concentrations (0.2–0.4 mg/mL).

The PC2 was positively correlated with lipase inhibition (0.9757) at the highest concentration (1 mg/mL) of the studied compounds and antibacterial activity against *K. pneumoniae* (0.5160). However, PC2 inversely contributed to antibacterial activity against the Gram-negative strain of *P. aeruginosa* (−0.6638), cytotoxic activity (−0.6998 to −0.9679) at moderate concentrations (0.4–0.8 mg/mL), and α-amylase inhibition (−0.7307 to −0.9125) at higher amidrazone concentrations (0.6–1 mg/mL).

As can be seen in Figure 9, compounds **2b**, **2c**, **2e**, and **2g** (with low antimicrobial and antioxidant activity, high cytotoxic activity, high α-amylase inhibition at low concentrations, and low lipase and pepsin inhibitions) were located to the left in the score plot having negative values for PC1, while the **2a**, **2d** and **2f** ones (with high antimicrobial and antioxidant activity, low cytotoxic activity, low α-amylase inhibition at large concentrations, and high lipase and pepsin inhibitions) were situated to the right in the diagram having positive values for PC1. Then, compounds **2a**, **2b**, and **2c** with low activity against Gram-negative bacteria (*E. coli*, *Y. enterocolitica*, *K. pneumoniae*, *P. aeruginosa*), low cytotoxic activity, and low α-amylase inhibition were located above the A1 axis, whereas **2d**, **2e**, **2f,** and **2g** derivatives with high cytotoxic activity and high α-amylase inhibition were identified under the A1 axis.

It is noteworthy that amidrazones **2a**–**2g** fell into three distinct groups (Figure 9). Three compounds (**2b**, **2c**, and **2a**) with low antibacterial activity, cytotoxicity, and α-amylase inhibition, moderate antifungal efficiency, and high lipase inhibition created an evidently distinct cluster. However, with the highest antioxidant potential (DPPH = 93.27% and FRAP = 5.79 mM), **2a** was distinct from **2b** and **2c**, although these three amidrazones were generally close to one another. Furthermore, another group of three compounds **2e**, **2f**, and **2g** with low antioxidant properties (DPPH = 83.34–89.33%, FRAP = 1.01–3.62 mM) and cytotoxic activity (10.3–12.4%) at the highest concentration (1 mg/mL), high α-amylase inhibition (32.4–83.3%), and moderate lipase (29.0–53.0%) and pepsin (36.8–73.6%) inhibition was clearly separated on the score plot. Interestingly, **2f** with the lowest cytotoxic activity (10.3%) was located some distance away from **2e** and **2g**. Finally, **2d** with the longest distance from all other amidrazones revealed the highest antibacterial and antifungal activity (as already mentioned in Section 2.3.4), high antioxidant potential and α-amylase inhibition at the highest concentration (1 mg/mL), and high lipase and pepsin inhibition at low concentrations. The reason for its different characteristics may be the presence of the -NO_2_ side group in the Ar^2^ substituent.

#### 2.4.2. Hierarchical Cluster Analysis

**Figure 10:**
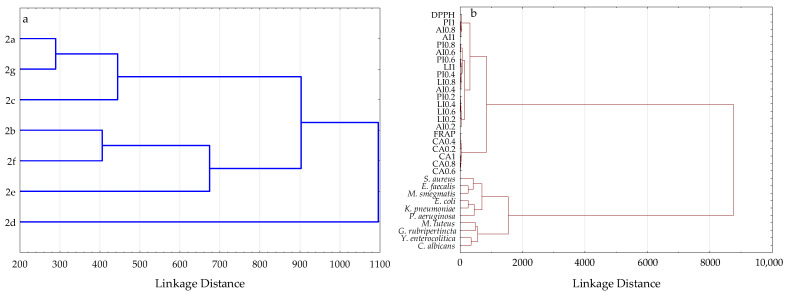
Dendrograms of hierarchical cluster analysis for (**a**) seven amidrazones **2a**–**2g** and (**b**) the studied variables. Abbreviations: DPPH—2,2-diphenyl-1-picrylhydrazyl method. FRAP—ferric reducing antioxidant power. AI0.2; AI0.4, AI0.6, AI0.8, and AI1; LI0.2, LI0.4, LI0.6, LI0.8, and LI1; PI0.2, PI0.4, PI0.6, PI0.8, and PI1; CA0.2, CA0.4, CA0.6, CA0.8, and CA1—α-amylase inhibition (AI), lipase inhibition (LI), and pepsin inhibition (PI), and cytotoxic activity (CA), respectively, by different concentrations of amidrazones (0.2–1 mg/mL).

The HCA was applied for the same reason as PCA, to group the synthesized amidrazones per their similarities and discrepancies based on the thirty-two investigated biological variables. The results obtained following HCA were presented as dendrograms (Figure 10), in which two well-defined clusters of seven compounds (Figure 10a) and two clusters of variable sets were visible (Figure 10b).

The first cluster included an inter-cluster of **2a**, **2g**, and **2c**, whereas **2b**, **2f**, and **2e** were arranged in the second inter-cluster. The inter-cluster, including **2a**, **2g**, and **2c**, was quite separated, because these compounds had the same moderate antifungal activity, low antibacterial and cytotoxic activity, and similar lipase and pepsin inhibitions. However, **2a** and **2g**, with insignificant differences in lipase inhibition, as well as the highest and lowest antioxidant properties (DPPH = 93.27% and 83.34%, respectively; FRAP = 5.79 mM and 1.01 mM, respectively), formed the sub-cluster. On the other hand, clustered compounds **2b** and **2f** exhibited the same low antimicrobial activity against Gram-negative bacteria, cytotoxicity, and high lipase inhibition at the lowest concentration (0.2 mg/mL). Meanwhile, **2e** and **2f** had similar DPPH values (89.33 and 88.61%, respectively) and enzyme inhibitions (α-amylase: 44.2–80.0% and 32.4–83.3%; lipase: 31.0–50.0% and 35.0–53.0%; pepsin: 38.8–73.2% and 38.3–73.6%). Additionally, the dendrogram presented in Figure 10a clearly separated amidrazone **2d**, which revealed the highest antimicrobial effect against Gram-positive and fungal strains, as well as high antioxidant activity, cytotoxicity, lipase and pepsin inhibitory activity at low concentrations, and α-amylase inhibition at the highest concentration.

**Figure 11:**
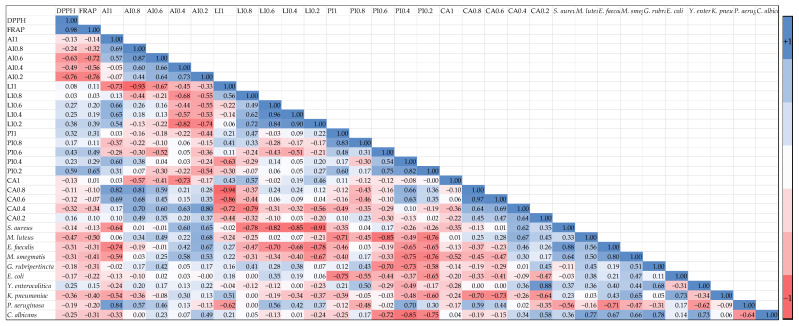
Correlation color matrix between biological parameters of seven amidrazones **2a**–**2g**. Abbreviations: DPPH—2,2-diphenyl-1-picrylhydrazyl method; FRAP—ferric reducing antioxidant power; AI0.2, AI0.4, AI0.6, AI0.8, and AI1; LI0.2, LI0.4, LI0.6, LI0.8, and LI1; PI0.2, PI0.4, PI0.6, PI0.8, and PI1; CA0.2, CA0.4, CA0.6, CA0.8, and CA1—α-amylase inhibition (AI), lipase inhibition (LI), pepsin inhibition (PI), and cytotoxic activity (CA), respectively, by different concentrations of amidrazones (0.2–1 mg/mL). Blue color indicates positive correlation, while red color signifies negative correlation.

Furthermore, HCA was utilized to group the thirty-two variables with common characteristics into clusters. A cluster composed of antioxidant potential, inhibition of three enzymes, and cytotoxic activity of the investigated compounds was clearly discernible, whereas their antibacterial and antifungal features formed the second cluster (Figure 10b). Meanwhile, the first group, exhibiting antioxidant capacity, enzyme inhibition, and cytotoxic activity, underwent further subdivision into two sub-groups, including the following: (I) DPPH; α-amylase, pepsin, and lipase inhibitory activity; and (II) FRAP and cytotoxicity at different concentrations of the investigated compounds. This can be explained by the fact that the antioxidant potential affected the efficiency of enzyme inhibition. However, the second cluster was classified into two sub-groups, consisting of inhibitory effect against (I) three Gram-positive bacteria strains (*S. aureus*, *E. faecalis*, *M. smegmatis*) and three Gram-negative bacteria strains (*E. coli*, *K. pneumoniae*, *P. aeruginosa*), as well as (II) two Gram-positive bacteria strains (*M. luteus*, *G. rubripertincta*), one Gram-negative bacteria strain (*Y. enterocolitica*), and the only fungal strain (*C. albicans*). The formation of inter-clusters indicates high relationships between these variables (color matrix presented in Figure 11).

The unsupervised methods, PCA and HCA, revealed that amidrazone **2d** bearing two electron-withdrawing substituents at Ar^1^ and Ar^2^ (2-pyridyl and 4-nitrophenyl), which is well-separated from the other synthesized derivatives, exhibited high antimicrobial, antioxidant, cytotoxic, and enzyme inhibitory activity. Apparently, replacing the 4-nitrophenyl substituent with a moderate electron-withdrawing 2-pyridyl at the Ar^2^ position in compound **2e** resulted in a slight decrease in its biological properties and a longer distance from the inter-cluster in the HCA dendrogram (Figure 10a). However, two electron-donating phenyl groups at positions Ar^1^ and Ar^2^ in compound **2a**, situated on the upper right quarter of the score plot (Figure 9a), were involved in the most effective scavenging of DPPH radicals and reduction in Fe(III)-TPTZ complex.

The PCA and HCA results together have shown that the analytical methods used to determine the biological properties of the synthesized amidrazone derivatives are different. In addition, the clustering behavior of these compounds appears to distinguish between the HCA and the PCA visualizations. Probably, the HCA may capture biological similarities more efficiently from a visual perspective than PCA because only the first two PCs, PC1 and PC2, are used on the score and loading plots.

#### 2.4.3. Correlation Analysis

In order to evaluate the strength and direction of the relationships among multiple variables, such as antioxidant properties, inhibitory activity against three enzymes, antibacterial, antifungal, and cytotoxic activities of seven amidrazones **2a**–**2g**, Pearson correlation analysis was carried out, with the obtained correlation coefficients being visualized on the color matrix (Figure 11).

As illustrated in Figure 11, correlation coefficients ranged from +1 (dark blue color) to −1 (dark red color), where positive values indicate a direct relationship and negative values indicate an inverse relationship. Expectantly, the color matrix illustrated significant positive relationship (r = 0.98) between the antioxidant activity measured by DPPH and FRAP assays. This high value of correlation coefficient confirms that, at the same time, all investigated amidrazones were capable of scavenging DPPH radicals and reducing (Fe^3+^-TPTZ) to an intense blue color (Fe^2+^-TPTZ) complex. The Pearson analysis also revealed strong negative correlations (r = −0.49 to −0.76) between antioxidant activity determined by DPPH and FRAP methods and α-amylase inhibition at low amidrazone concentrations (0.2–0.6 mg/mL), whereas DPPH and FRAP were positively correlated (r = 0.59–0.65) with pepsin inhibition at the lowest concentration of the studied derivatives, suggesting that enzyme inhibition was linked to their antioxidant activity. Meanwhile, positive correlations were observed between cytotoxic activity (r = 0.45–0.97) and enzyme inhibition (r = 0.31–0.96) at the increased concentrations. This indicates that a higher concentration of each amidrazone caused stronger cytotoxic and enzyme inhibition effects. Interestingly, lipase inhibition at low amidrazone concentrations positively correlated (r = 0.54–0.66) with α-amylase inhibition at the highest concentration, while negative associations (r = −0.33 to −0.82) were observed between inhibition potential of derivatives at 0.2 and 0.4 mg/mL against lipase and α-amylase. Moreover, positive correlation coefficients were found between the inhibition of α-amylase by amidrazones at low concentrations and the growth of four Gram-positive bacteria (r = 0.42–0.68) and cytotoxic activity (r = 0.35–0.81). On the contrary, pepsin inhibition at low amidrazone concentrations inversely correlated (r = −0.33 to −0.85) with the concerned bacterial and fungal strains (except *S. aureus*, *Y. enterocolitica*, and *P. aeruginosa*). However, *S. aureus* and *E. faecalis* had strong negative correlations (r = −0.47 to −0.91) with lipase inhibition. Depending on concentrations, the discussed compounds could inhibit these bacterial strains, but did not exhibit pepsin and lipase inhibition. Most notably, positive correlations between Gram-positive bacteria strains were observed (r = 0.33–0.88). Moreover, positive linear associations were found between each Gram-positive bacterial strain and antifungal efficacy (r = 0.36–0.78). These correlation coefficients suggest that the investigated amidrazones had a similar trend for *S. aureus*, *M. luteus*, *E. faecalis*, *M. smegmatis*, *G. rubripertincta*, and *C. albicans*. In contrast, the Gram-negative bacterium *P. aeruginosa* revealed moderate negative relationships with two Gram-positive bacteria, *S. aureus* (r = −0.56) and *E. faecalis* (r = −0.71), as well as with the Gram-negative bacterium *Y. enterocolitica O3* (r = −0.62) and the fungus *C. albicans* (r = −0.64). Therefore, the compounds with antimicrobial activity against *P. aeruginosa* caused no activity against *S. aureus*, *E. faecalis*, and *C. albicans*.

## 3. Materials and Methods

### 3.1. General Information

Reagents and solvents were acquired from Sigma-Aldrich or Avantor Performance Materials Poland. Melting points were measured using a Mel-Temp apparatus (Electrothermal, Stone, UK). ^1^H and ^13^C NMR spectra (including ^1^H-^13^C HMQC and HMBC) as well as ^1^H-^15^N HMBC-NMR spectra were recorded in DMSO-d_6_ at 295–300 K by Bruker Avance III 400 MHz or Bruker Avance III 700 MHz NMR spectrometers (Bruker Corporation, Billerica, MA, USA). The ^1^H and ^13^C chemical shifts were referenced to TMS, with residual ^1^H and ^13^C solvent signals as primary references (DMSO-d_6_: 2.50 ppm and 40.0 ppm, respectively), whereas the ^15^N chemical shifts were referenced to external neat nitromethane. High-resolution mass spectra (HRMS) were performed on a Synapt G2-Si mass spectrometer (Waters Corporation, Milford, MA, USA). The course of reactions was monitored using TLC chromatography (silica gel on TLC-PET foils, Sigma-Aldrich, St. Louis, MO, USA).

### 3.2. Synthesis of Amidrazones ***2a***–***2g***

Thioamides **1a**–**1g** were obtained according to the previously described methods: **1a** [56], **1b**–**1d** [57], **1e** [58], **1f**–**1g** [59]. Amidrazones **2a**–**2g** were obtained by the modified Spassov method [35] in the reactions of 0.01 mol of thioamides **1a**–**1g** with an excess of 64% hydrazine hydrate solution (0.02–0.10 mol). Unless otherwise stated below, the reaction was carried out at room temperature for 24 h (except for compounds **2c**–**2e**), then 20 mL of water was added. The resulting precipitate was filtered off and washed with water, followed by purification by crystallization (except for **2c**).

The detailed reaction and purification conditions for **2a**–**2g** are given below, while their HRMS are presented in the Supplementary Data (Part C—Figures S13–S19).

- *N*-phenylbenzenecarbohydrazonamide (**2a**)

For **2a**, 2.13 g (0.01 mol) of *N*-phenylbenzenecarbothioamide **1a** was dissolved in 5 mL of anhydrous ethanol with gentle heating, then 1 mL (0.02 mol) of 64% hydrazine was added. After 24 h, 20 mL of water was added. After 5 min, obtained precipitate was filtered off and recrystallized from 10 mL of ethanol. After adding 30 mL of water to the crystallizer, a pinkish crystalline precipitate was formed, which was filtered off. Yield 46.97%, m.p. 86–88 °C. HR-MS *m*/*z*: 212.1184 [M^+^ + 1] (calculated for 212.1188: C_13_H_14_N_3_).

- *N*-phenylpyridine-2-carbohydrazonamide (**2b**)

For **2b**, 2.14 g (0.01 mol) of *N*-phenylpyridine-2-carbothioamide **1b** was mixed with 5 mL (0.1 mol) of 64% hydrazine. After 24 h, 20 mL of water was added, the precipitate filtered off, and recrystallized from methanol. Yield 47.41%, m.p. 109–112 °C. HR-MS *m*/*z*: 213.1136 [M^+^ + 1] (calculated for 213.1140: C_12_H_13_N_4_).

- *N*-(4-methylphenyl)pyridine-2-carbohydrazonamide (**2c**)

For **2c**, 2.28 g (0.01 mol) of *N*-(4-methylphenyl)pyridine-2-carbothioamide **1c** was mixed with 1.5 mL (0.03 mol) 64% hydrazine. The solution was left for 3 days at room temperature. Then 20 mL of water was added, and the precipitate was filtered off. The crude solid was dissolved in 20 mL of methanol, and the insoluble precipitate was filtered off and discarded. Then water was gradually added to the methanol solution, the solution was decanted from the precipitate, and water was added again. The operation was repeated as long as a precipitate was formed. Fractions with a melting point below 74 °C were purified in this way again. Yield 30.16%, m.p. 75–78 °C. HR-MS *m*/*z*: 227.1290 [M^+^ + 1] (calculated for 227.1297: C_13_H_15_N_4_).

- *N*-(4-nitrophenyl)pyridine-2-carbohydrazonamide (**2d**)

For **2d**, 2.59 g (0.01 mol) *N*-(4-nitrophenyl)pyridine-2-carbothioamide **1d** was mixed with 10 mL 96% ethanol and 6 mL (0.12 mol) 64% hydrazine. It was heated lightly for 1–2 min until the precipitate was dissolved, then left at room temperature. After 1 h, 20 mL of water was added to the mixture. The obtained light yellow precipitate was filtered off and recrystallized from ethanol. Yield 52.32%, m.p. 174–177 °C. HR-MS *m*/*z*: 258.0986 [M^+^ + 1] (calculated for 258.0991: C_12_H_12_N_5_O_2_).

- *N*-(pyridin-2-yl)pyridine-2-carbohydrazonamide (**2e**)

For **2e**, 2.15 g (0.01 mol) of *N*-(pyridin-2-yl)pyridine-2-carbothioamide **1e** was mixed with 3 mL (0.06 mol) of 64% hydrazine. After 24 h, 20 mL of water was added. The precipitate was filtered and recrystallized from 50 mL of water with the addition of about 2.5 g of activated carbon (about 20 min of heating). Yield 47.47%, m.p. 107–110 °C. HR-MS *m*/*z*: 214.1086 [M^+^ + 1] (calculated for 214.1093: C_11_H_12_N_5_).

- *N*-phenylpyridine-4-carbohydrazonamide (**2f**)

For **2f**, 2.14 g (0.01 mol) of *N*-phenylpyridine-4-carbothioamide **1f** was mixed with 5 mL (0.1 mol) of 64% hydrazine. After 24 h, 20 mL of water was added. The obtained precipitate was filtered off and purified by crystallization from a mixture of water and methanol (1:1). Yield 56.06%, m.p. 125–128 °C. HR-MS *m*/*z*: 213.1136 [M^+^ + 1] (calculated for 213.1140: C_12_H_13_N_4_).

- *N*-(4-methylphenyl)pyridine-4-carbohydrazonamide (**2g**)

For **2g**, 2.28 g (0.01 mol) of *N*-(4-methylphenyl)pyridine-4-carbothioamide **1g** was mixed with 5 mL (0.1 mol) of 64% hydrazine. After 24 h, 20 mL of water was added. The obtained precipitate was filtered off and purified by crystallization from a mixture of water and methanol (1:1). Yield 33.61%, m.p. 127–129 °C. HR-MS *m*/*z*: 227.1295 [M^+^ + 1] (calculated for 227.1297: C_13_H_15_N_4_).

### 3.3. Antioxidant Activity of the Synthesized Amidrazones ***2a***–***2g***

Antioxidant activity of **2a**–**2g** was tested using two alternative analytical methods: DPPH and FRAP.

#### 3.3.1. DPPH Assay

This method involves a reduction of the stable, purple-colored 2,2-diphenyl-1-picrylhydrazyl (DPPH) radical in the reaction with a given antioxidant [60]. The DPPH solution was prepared by dissolving 7.89 mg of DPPH in 100 mL of 99.5% ethanol and stored in darkness for 2 h. Then, 800 µL of Tris-HCl buffer (pH 7.4) was mixed with 1000 µL of DPPH solution in an Eppendorf tube, followed by the addition of 200 µL of the **2a**–**2g** sample (1 mg/mL). The mixture was incubated at room temperature for 30 min, and absorbance was measured at 517 nm. A blank was prepared using 1200 µL of ethanol and 800 µL of Tris-HCl buffer. The percentage of DPPH radical scavenging activity was calculated using the formula:

% DPPH Scavenging = [(Abs__control_ − Abs__sample_)/Abs__control_] × 100 (1)

#### 3.3.2. FRAP Assay

The FRAP assay evaluates antioxidant capacity by measuring reduction in the ferric Fe^3+^-TPTZ complex to the respective ferrous Fe^2+^-TPTZ species (TPTZ being 2,4,6-tris(pyrid-2-yl)-1,3,5-triazine), which results in a navy blue color [60]. To prepare the reagents, sodium acetate trihydrate (3.1 g) was dissolved and combined with 16 mL glacial acetic acid to make an acetic acid buffer (pH 3.6). Separately, 0.0256 g of TPTZ was dissolved in 4 mL of 40 mM HCl, and 0.276 g of FeCl_3_·6H_2_O was dissolved in water, then diluted to 50 mL. The FRAP working solution was prepared by mixing the acetic acid buffer and the FeCl_3_-TPTZ solutions in a 10:1 ratio, then the solution was incubated at 37 °C. For the assay, 50 µL of FRAP working solution was mixed with 950 µL of the **2a**–**2g** working solutions (obtaining final concentrations of amidrazones **2a**–**2g** of 4 mM), incubated in darkness at 37 °C for 15 min, and absorbance was measured at 593 nm. FeSO_4_ served as the standard, and distilled water was used as the blank. The antioxidant capacity was calculated using the formula:

FRAP = (Abs__sample_ − Abs__blank_)/Abs__standard_ × standard concentration (2)

### 3.4. Enzymes Inhibitory Activity of the Synthesized Amidrazones ***2a***–***2g***

#### 3.4.1. α-Amylase Inhibition

The α-Amylase inhibitory assay was determined by the Sundaram method [61]. Briefly, 250 µL of **2a**–**2g** solution was placed in a tube, and then 250 µL of 0.02 M sodium phosphate buffer with pH 6.9 and α-amylase solution (0.5 mg/mL) were added. The mixture was incubated for 10 min at 25 °C, then 250 µL of 1% starch solution as substrate (prepared in 0.02 M sodium phosphate buffer with pH 6.9) was added, and then further incubated for 10 min at 37 °C. By adding 500 µL of dinitrosalicylic acid, the reaction was terminated. Finally, each reaction mixture was diluted with 5 mL of water, and the absorbance was measured at 540 nm. The control was free DMSO. The α-amylase inhibitory activity was calculated as a percentage inhibition:

% Inhibition = [(Abs__control_ − Abs__sample_)/Abs__control_] × 100 (3)

#### 3.4.2. Lipase Inhibition

The lipase inhibitory assay was determined by the Sikander method [62]. The assay is based on a change in pH from neutral to acidic after lipase activity, which changes the color of the medium. Plates were prepared by using the medium composed of 2% agar (*w*/*v*) along with 2.5% (*v*/*v*) olive oil and 1% (*w*/*v*) phenol red as an indicator. Lipase solution was prepared in 100 mM Tris-HCl buffer (30 mg/mL). After this, 60 μL of the master mix was prepared using 30 μL of lipase solution and 30 μL of **2a**–**2g** sample solutions and poured into wells (obtaining final concentrations of 0.2 mg/mL, 0.4 mg/mL, 0.6 mg/mL, 0.8 mg/mL, and 1 mg/mL). The control well contained 30 μL of lipase and buffer. The plates were incubated for 24 h at 37 °C. After incubation, the change in color in the control (a yellow zone around the well of a red background due to acidic pH) indicated the activity of lipase. Lipase inhibitory activity was calculated as percentage inhibition:

% Inhibition = [(Zone__control_ − Zone__sample_)/Zone__control_] × 100 (4)

#### 3.4.3. Pepsin Inhibition

The pepsin inhibitory assay was determined by the Mushtaq method [63]. Briefly, 100 µL of **2a**–**2g** solution was mixed with 100 µL of pepsin solution (5 mg in 0.01 M HCl) and incubated at room temperature for 30 min to allow for enzyme activity. Then, 200 µL of albumin solution (5 mg/mL) was added as a substrate, and the mixture was further incubated for 20 min. To stop the reaction, 1600 µL of biuret reagent was added. Absorbance was measured at 540 nm. Sucralfate and DMSO were used as positive and negative controls, respectively. Pepsin inhibitory activity was calculated using the formula:

% Inhibition = [(Abs__positive control_ − Abs__sample_)/(Abs__positive control_ − A__negative control_)] × 100 (5)

### 3.5. Cytotoxic Activity of the Synthesized Amidrazones ***2a***–***2g***

The cytotoxicity of **2a**–**2g** was determined by examination of the level of erythrocyte hemolysis using the Maqsood method [64]. The **2a**–**2g** compounds were dissolved in DMSO. Fresh blood (5 mL) was centrifuged, and red blood cells were washed with phosphate buffer (pH 7.4). Then, 20 µL of the **2a**–**2g** sample was mixed with 180 µL of diluted blood cell suspension and incubated at 37 °C for 35 min, with shaking after 10 min. Following incubation, the mixture was chilled on ice, centrifuged, and 100 µL of the supernatant was diluted with 900 µL PBS. From each tube, 200 µL was transferred to an ELISA plate. Triton X-100 (0.1%) served as the positive control, and PBS as the negative control. Absorbance was measured at 576 nm using an ELISA reader (Elx 800, BioTek, San Diego, CA, USA), and the percentage of hemolysis was calculated using the formula:

% Hemolysis = [(Abs__sample_ − Abs__negative control_)/Abs__positive control_] × 100 (6)

### 3.6. Antimicrobial Activity of the Synthesized Amidrazones ***2a***–***2g***

#### 3.6.1. Disk Diffusion Method

To preliminarily determine the sensitivity of the tested strains to the derivatives, the compounds’ activity was tested using the disk diffusion method according to the European Committee on Antimicrobial Susceptibility Testing [65].

The turbidity of the cultures was set to 0.5 McFarland units, and 100 μL of the culture was applied to plates containing MH agar medium. Then, 10 μL of the tested compounds dissolved in DMSO at a concentration of 10 mg/mL (yielding 100 μg/disk) were applied onto sterile 6 mm diameter filter paper disks (BioMaxima, Lublin, Poland) and dried at room temperature. The disks with the tested compounds were then placed on the seeded plates. As controls, DMSO (10 μL) and filter paper disks containing defined concentrations of the antibiotics ampicillin (10 μg), gentamicin (10 μg), or fluconazole (25 μg) (BioMaxima, Lublin, Poland) were used. The plates were incubated at 37 °C for 18 h.

Compounds that inhibited microbial growth by at least 8 mm were selected for MIC determination by the microdilution method.

#### 3.6.2. Determination of the Minimum Inhibitory Concentration (MIC)

The antimicrobial activity of **2a**–**2g** was determined by the broth microdilution method, according to the Clinical and Laboratory Standards Institute (CLSI) guidelines [66]. Each compound was dissolved in DMSO to a concentration of 10.24 mg/mL, then diluted in Mueller-Hinton broth (MH), and serial two-fold dilutions were performed (ranging from 512 to 0.5 μg/mL) in a 96-well microplate.

In order to determine the antibacterial properties of **2a**–**2g**, the following strains of bacteria were chosen: Gram-positive (pathogenic *S. aureus* ATCC 25923, *M. luteus* ATCC 4698, *E. faecalis* ATCC 29212, and non-pathogenic *M. smegmatis*, *Gordonia rubripertincta*) and Gram-negative (drug-sensitive *E. coli* ATCC 25922, *Y. enterocolitica* serotype O3, and drug-resistant *K. pneumoniae* ATCC 700603 and *P. aeruginosa* ATCC 27853). Antifungal properties of **2a**–**2g** were tested on the pathogenic yeast *C. albicans* ATCC 90028.

The MIC value was defined as the lowest concentration of the compound at which no bacterial growth was observed. The optical density was measured spectrophotometrically at 550 nm; the control was a bacterial culture without the addition of the amidrazones.

#### 3.6.3. Atomic Force Microscopy (AFM) Analysis

Amidrazone **2d**, which exhibited the best antimicrobial potency, was selected for analysis of activity against *S. aureus* ATCC 25923, *M. smegmatis*, and *C. albicans* ATCC 90028 using atomic force microscopy (AFM). Briefly, **2d** was dissolved in DMSO to a concentration of 6.4 mg/mL and added to MH broth to obtain the ½ MIC value specific for each strain. Then, the tested microorganisms’ culture, corresponding to 0.5 on the McFarland scale, was added. The control was a culture prepared in the same way, but without the addition of **2d**. The cultures were incubated at 37 °C for 48 h and centrifuged for 5 min at 14,500 rpm. The bacterial pellet was washed first with sterile saline, then twice with deionized water. After the final centrifugation step, the bacteria were suspended in 5 μL of sterile water and applied to the surface of mica disks, left to dry overnight at room temperature. The surface of the cells was measured using NanoScope V AFM in the Peak-Force Quantitative Nanomechanical Mapping Mode (Bruker, Veeco Instruments Inc., Billerica, MA, USA) and NanoScope 8.15 software.

### 3.7. Statistical Analysis

All the experiments for the biological properties of the synthesized amidrazones were performed in triplicate, and the results were expressed as mean (c) ± standard error of the mean (SEM). The principal component analysis (PCA) was utilized as a data reduction technique to generate a visual plot of the synthesized amidrazone derivatives and their distribution on a score plot and to visualize potential relationships among their biological properties. However, the hierarchical cluster analysis (HCA) was performed using the Ward clustering algorithm with squared Euclidean distance to sort the investigated amidrazones into groups. Moreover, to analyze the relationships among the biological features of amidrazones, Pearson correlations were calculated and depicted as a correlation color matrix.

The statistical analysis was carried out using Statistica 8.0 software (StatSoft Inc., Tulsa, OK, USA).

## 4. Conclusions

Seven *N*^3^-substituted amidrazones **2a**–**2g** containing aryl groups such as phenyl, 4-methylphenyl, 4-nitrophenyl, 2-pyridyl, and/or 4-pyridyl, were synthesized and their molecular structures confirmed using ^1^H, ^13^C, and ^15^N NMR spectroscopy. The evaluation of biological properties revealed that all synthesized compounds exhibited antioxidant activity, inhibitory activity against α-amylase, lipase, and pepsin, cytotoxic activity, as well as antibacterial and antifungal activity. Interestingly, two amidrazones, **2a**, with two phenyl groups in Ar^1^ and Ar^2^ positions, and **2d**, substituted by 2-pyridyl and 4-nitrophenyl in these positions, had the highest ability to react with the DPPH radical and were capable of reducing the Fe^3+^-TPTZ complex. In addition, **2d** amidrazone had potential inhibitory activity against α-amylase at the highest concentration (1 mg/mL) and lipase and pepsin at the lowest concentration (0.2 mg/mL). Then, **2b** and **2d** (with 2-pyridyl as Ar^1^ and phenyl or 4-nitrophenyl as Ar^2^) at the highest concentration had vigorous cytotoxic activity, whereas the cytotoxic effect of all **2a**–**2g** compounds at lower concentrations was negligible. Moreover, **2d** exhibited the most potent antibacterial activity against all Gram-positive bacterial strains and the antifungal activity, whereas it was much less effective against Gram-negative bacterial strains. The varying sensitivity of the tested microorganisms to **2d** reflects noticeable differences in cell surface properties. The most probable reason for the increase in surface roughness is the rupture of bacterial and fungal cells due to the amidrazone binding.

The chemometric analysis conducted for this study confirmed significant differences in the biological activity of **2a**–**2g**, depending on the Ar^1^ and Ar^2^ substituents. At the same time, PCA and HCA displayed some common biological properties among these amidrazones, allowing for them to be grouped into characteristic clusters. This may indicate a future biological use of amidrazones, and not just as building blocks for synthesis, as they have been used so far.

## Figures and Tables

**Table 1:** Ar^1^ and Ar^2^ substituents in amidrazones **2a**–**2g**.

	2a	2b	2c	2d	2e	2f	2g
Ar^1^	C_6_H_5_	2-C_5_H_4_N	2-C_5_H_4_N	2-C_5_H_4_N	2-C_5_H_4_N	4-C_5_H_4_N	4-C_5_H_4_N
Ar^2^	C_6_H_5_	C_6_H_5_	4-CH_3_-C_6_H_4_	4-NO_2_-C_6_H_4_	2-C_5_H_4_N	C_6_H_5_	4-CH_3_-C_6_H_4_

**Table 2:** ^1^H NMR chemical shifts in amidrazones **2a**–**2g** (in DMSO-d_6_).

Compound	Ar^1^, Ar^2^	H1 ^a^	H3 ^b^	H2′	H3′	H4′	H5′	H6′	H2″	H3″	H4″	H5″	H6″
**2a** [13]	C_6_H_5_, C_6_H_5_	6.15	7.74	7.52	7.30	7.25	#	##	6.56	7.10	6.70	###	####
**2b** [13]	2-C_5_H_4_N, C_6_H_5_	6.23	7.82	*	8.46	7.27	7.77	7.91	6.62	7.14	6.74	###	####
**2c** [13]	2-C_5_H_4_N, 4-CH_3_-C_6_H_4_	6.12	7.72	*	8.45	7.27	7.77	7.90	6.53	6.95	* ^c^	###	####
**2d** [13]	2-C_5_H_4_N, 4-NO_2_-C_6_H_4_	6.80	8.86	*	8.45	7.27	7.79	7.90	6.63	8.05	*	###	####
**2e** [13]	2-C_5_H_4_N, 2-C_5_H_4_N	6.60	8.42	*	8.43	7.26	7.76	7.90	*	8.03	6.71	7.53	6.70
**2f** [13]	4-C_5_H_4_N, C_6_H_5_	6.74	7.74	7.42	8.46	*	#	##	6.53	7.12	6.71	###	####
**2g**	4-C_5_H_4_N, 4-CH_3_-C_6_H_4_	6.60	7.58	7.37	8.43	*	#	##	6.42	6.91	* ^d^	###	####

^a^ NH_2_ protons; ^b^ NH protons; ^c^ instead of H4″, the CH_3_ group at 4″ position appears at 2.18 ppm; ^d^ instead of H4″, the CH_3_ group at 4″ position appears at 2.14 ppm. *—no proton, #—like H3′, ##—like H2′, ###—like H3″, ####—like H2″.

**Table 3:** ^13^C NMR chemical shifts in amidrazones **2a**–**2g** (in DMSO-d6).

Compound	Ar^1^, Ar^2^	C2a	C1′	C2′	C3′	C4′	C5′	C6′	C1″	C2″	C3″	C4″	C5″	C6″
**2a** [13]	C_6_H_5_, C_6_H_5_	138.9	136.3	126.5	128.6	128.1	#	##	143.7	116.2	129.2	119.0	###	####
**2b** [13]	2-C_5_H_4_N, C_6_H_5_	138.1	153.9	*	148.6	123.0	136.9	120.4	142.5	117.0	128.9	119.5	###	####
**2c** [13]	2-C_5_H_4_N, 4-CH_3_-C_6_H_4_	138.4	153.8	*	148.5	123.0	136.9	120.4	139.8	117.3	129.3	128.3 ^a^	###	####
**2d** [13]	2-C_5_H_4_N, 4-NO_2_-C_6_H_4_	135.0	153.5	*	148.8	123.0	137.0	120.3	150.4	115.1	125.8	138.6	###	####
**2e** [13]	2-C_5_H_4_N, 2-C_5_H_4_N	136.9	154.1	*	148.4	122.9	136.8	120.5	155.7	*	147.8	114.9	137.8	110.9
**2f** [13]	4-C_5_H_4_N, C_6_H_5_	135.3	143.8	120.3	150.0	*	#	##	143.5	115.7	129.4	119.1	###	####
**2g**	4-C_5_H_4_N, 4-CH_3_-C_6_H_4_	136.0	143.8	120.5	150.0	*	#	##	140.9	116.1	129.8	127.9 ^b^	###	####

^a^ Additionally, the CH_3_ group at 4″ position appears at 20.6 ppm; ^b^ additionally, the CH_3_ group at 4″ position appears at 20.6 ppm. *—no carbon, #—like C3′, ##—like C2′, ###—like C3″, ####—like C2″.

**Table 4:** ^15^N NMR chemical shifts in amidrazones **2a**–**2g** (in DMSO-d6; referenced to external neat CH_3_NO_2_).

Compound	Ar^1^, Ar^2^	N1	N2	N3	N2′/N4′ in Ar^1^	N2″ in Ar^2^	NO_2_
**2a**	C_6_H_5_, C_6_H_5_	n.d.	n.d.	−296.7	*	*	*
**2b**	2-C_5_H_4_N, C_6_H_5_	−268.0	n.d.	−296.3	−74.3^N2′^	*	*
**2c**	2-C_5_H_4_N, 4-CH_3_-C_6_H_4_	n.d.	n.d.	−299.3	−76.9^N2′^	*	*
**2d**	2-C_5_H_4_N, 4-NO_2_-C_6_H_4_	n.d.	n.d.	−287.0	−74.4^N2′^	*	−8.8
**2e**	2-C_5_H_4_N, 2-C_5_H_4_N	n.d.	n.d.	−285.0	−76.8^N2′^	−108.9^N2″^	*
**2f**	4-C_5_H_4_N, C_6_H_5_	n.d.	n.d.	−301.2	−69.4^N4′^	*	*
**2g**	4-C_5_H_4_N, 4-CH_3_-C_6_H_4_	n.d.	n.d.	−301.7	−69.1^N4′^	*	*

*—No nitrogen.

**Table 5:** Antibacterial and antifungal activity of amidrazones **2a**–**2g** and the reference drugs, determined by broth microdilution method. Results are obtained from three independent experiments and presented as minimal inhibitory concentration (MIC, µg/mL). Gen—gentamycin; AmB—amphotericin B.

Strain	MIC [µg/mL]								
	2a	2b	2c	2d	2e	2f	2g	Gen	AmB
*S. aureus* ATCC 25923 ^a^	>512	256	>512	64	>512	128	512	1	-
*M. luteus* ATCC 4698 ^a^	256	512	128	128	512	256	512	2	-
*E. faecalis* ATCC 29212 ^a^	512	>512	512	128	>512	256	>512	16	-
*M. smegmatis* ^a^	512	512	512	128	512	512	512	64	-
*G. rubripertincta* ^a^	128	512	256	64	512	512	128	64	-
*E. coli* ATCC 25922 ^b^	512	512	256	256	256	512	512	1	-
*Y. enterocolitica* O3 ^b^	256	256	256	128	512	256	128	1	-
*K. pneumoniae* ATCC 700603 ^b^	512	512	512	256	256	>512	512	4	-
*P. aeruginosa* ^b^ ATCC 27853	512	512	512	>512	512	>512	512	2	-
*C. albicans* ^c^ ATCC 90028	128	256	128	32	256	128	128	-	0.125

^a^ Gram-positive bacteria, ^b^ Gram-negative bacteria, ^c^ fungus (yeast), (-)—not determined.

## Data Availability

The original contributions presented in this study are included in the article/Supplementary Materials. Further inquiries can be directed to the corresponding author.

## Supplementary Materials

The following supporting information can be downloaded at: https://www.mdpi.com/article/10.3390/ijms27020746/s1.

## Abbreviations

The following abbreviations are used in this manuscript:

ATTC	American Type Culture Collection
DMSO	Dimethyl sulfoxide
DPPH	2,2-diphenyl-1-picrylhydrazyl
HCA	Hierarchical cluster analysis
FRAP	Ferric reducing antioxidant power
HMBC	Heteronuclear multiple bond coherence
HMQC	Heteronuclear multiple quantum correlation
MIC	Minimal inhibitory concentration
PCA	Principal component analysis
SEM	Standard error of the mean
TPTZ	2,4,6-tris(2-pyridyl)-*s*-triazine
