# Supplementary material for: Spectroscopic Methods in Evaluation of Antioxidant Potential, Enzyme Inhibition, Cytotoxicity, and Antimicrobial Activity of the Synthesized N3-Substituted Amidrazones
Source: Int J Mol Sci. 2026 Jan 12;27(2):746. doi: 10.3390/ijms27020746 (PMC12841134; doi:10.3390/ijms27020746)
Supplement: Supplementary file 1 [file ijms-27-00746-s001.zip › ijms-4077052-supplementary.pdf]

Spectroscopic Methods in Evaluation of Antioxidant Potential, Enzyme Inhibition, Cytotoxicity, and Antimicrobial Activity of the Synthesized N^3-Substituted Amidrazones

Renata Paprocka [1], Leszek Pazderski [2], Jolanta Kutkowska [3], Iqra Naeem [4], Amna Shahid Awan [4], Zahid Mushtaq [4] and Aleksandra Szydłowska-Czerniak [2,*]

[1] Department of Organic Chemistry, Faculty of Pharmacy, Collegium Medicum in Bydgoszcz, Nicolaus Copernicus University in Toruń, Jurasza Str. 2, 85-089 Bydgoszcz, Poland; renata.bursa@cm.umk.pl

[2] Department of Analytical Chemistry and Applied Spectroscopy, Faculty of Chemistry, Nicolaus Copernicus University in Toruń, Gagarina Str. 7, 87-100 Toruń Poland; leszekp@chem.umk.pl

[3] Department of Genetics and Microbiology, Institute of Biological Sciences, Maria Curie-Skłodowska
University, Akademicka Str. 19, 20-033 Lublin, Poland; jolanta.kutkowska@mail.umcs.pl

[4] Bioactive Molecules Research Lab (BMRL), Department of Biochemistry, University of Agriculture Faisalabad, Faisalabad 38000, Pakistan; iqranaeem305@gmail.com (I.N.), awanamnashahid@gmail.com (A.S.A.), zahidmushtaquaf@uaf.edu.pk (Z.M)

* Correspondence: olasz@umk.pl (A.S.-C.)

PART A. ^{1}H, ^{13}C, ^{1}H-^{13}C HMQC, ^{1}H-^{13}C HMBC NMR spectra of amidrazone 2g

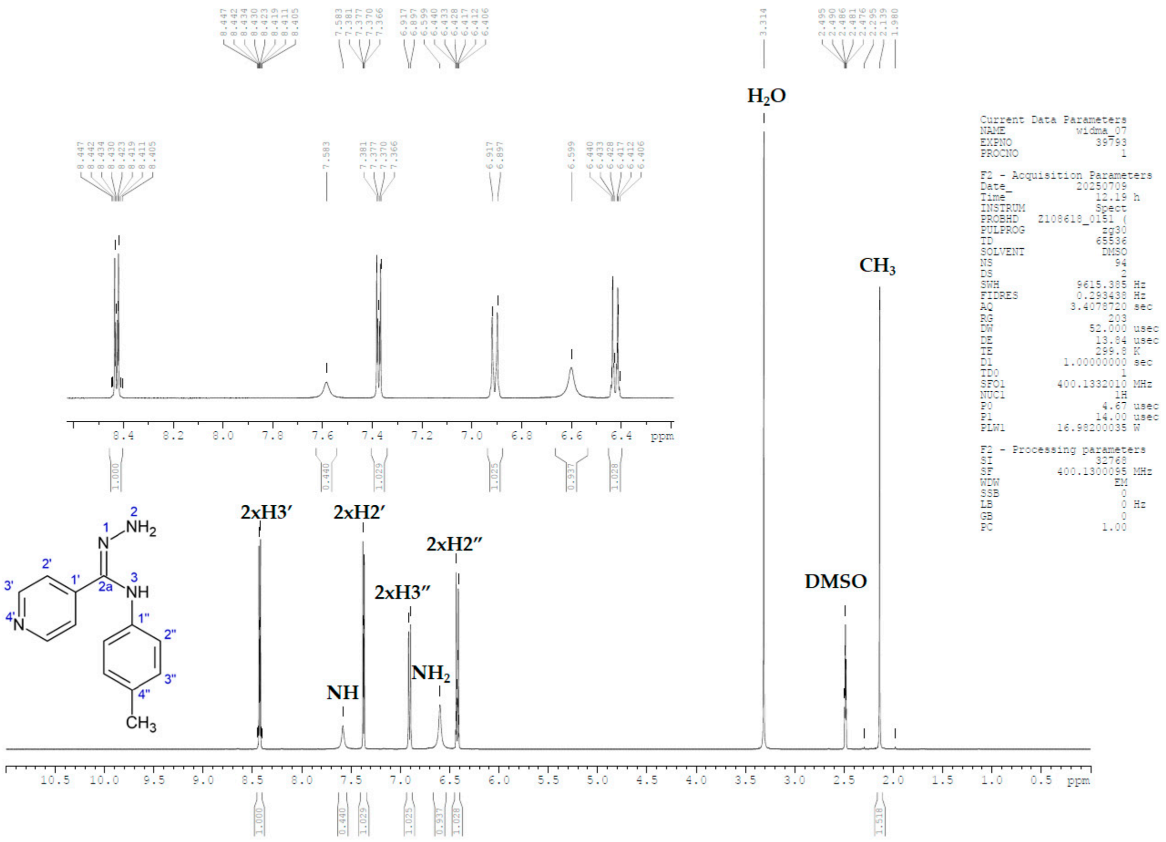

Figure S1. ^{1}H NMR spectrum of amidrazone **2g** (signals of H2′, H3′, H2″, and H3″ are twice the intensity as each derives from a given hydrogen atom and its equivalent counterpart in the second half of the 4-pyridyl or 4-methylphenyl ring).

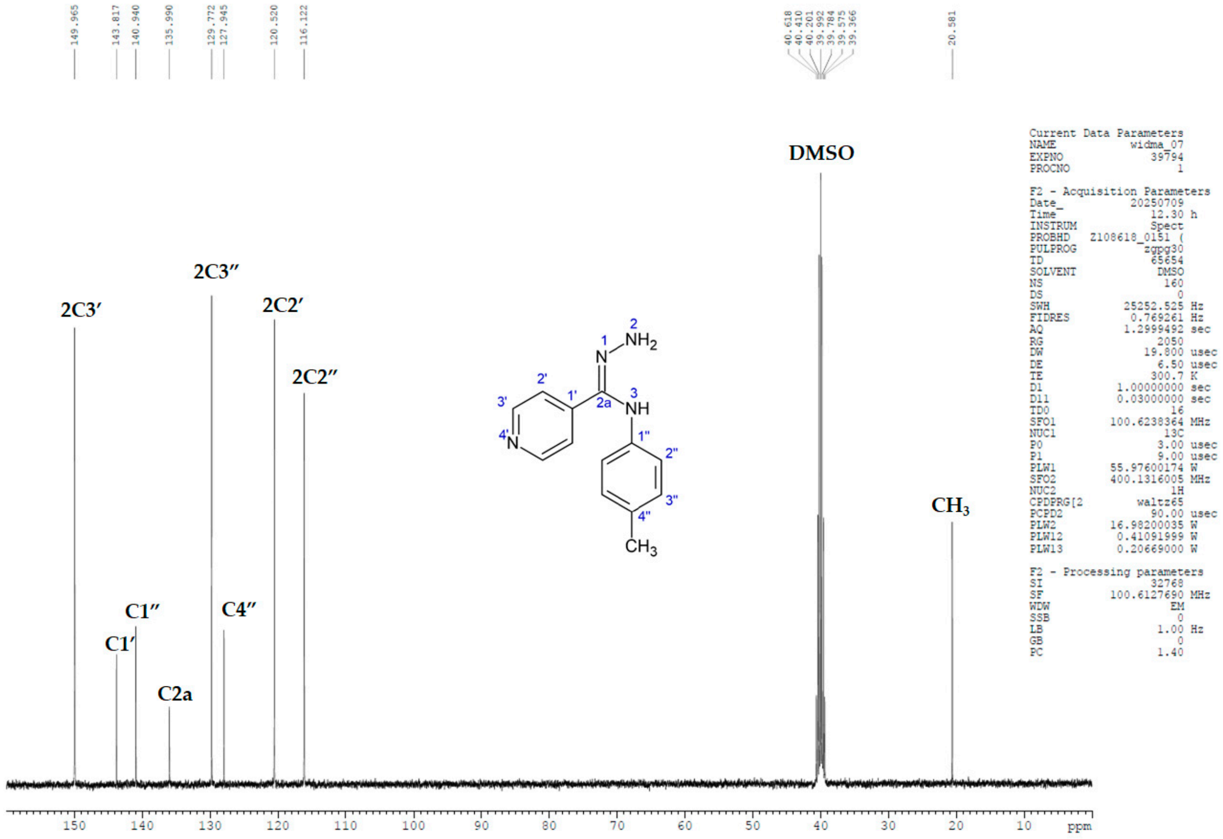

Figure S2. ^{13}C NMR spectrum of amidrazone **2g** (signals of C2′, C3′, C2″, and C3″ are twice the intensity as each derives from a given carbon atom and its equivalent counterpart in the second half of the 4-pyridyl or 4-methylphenyl ring).

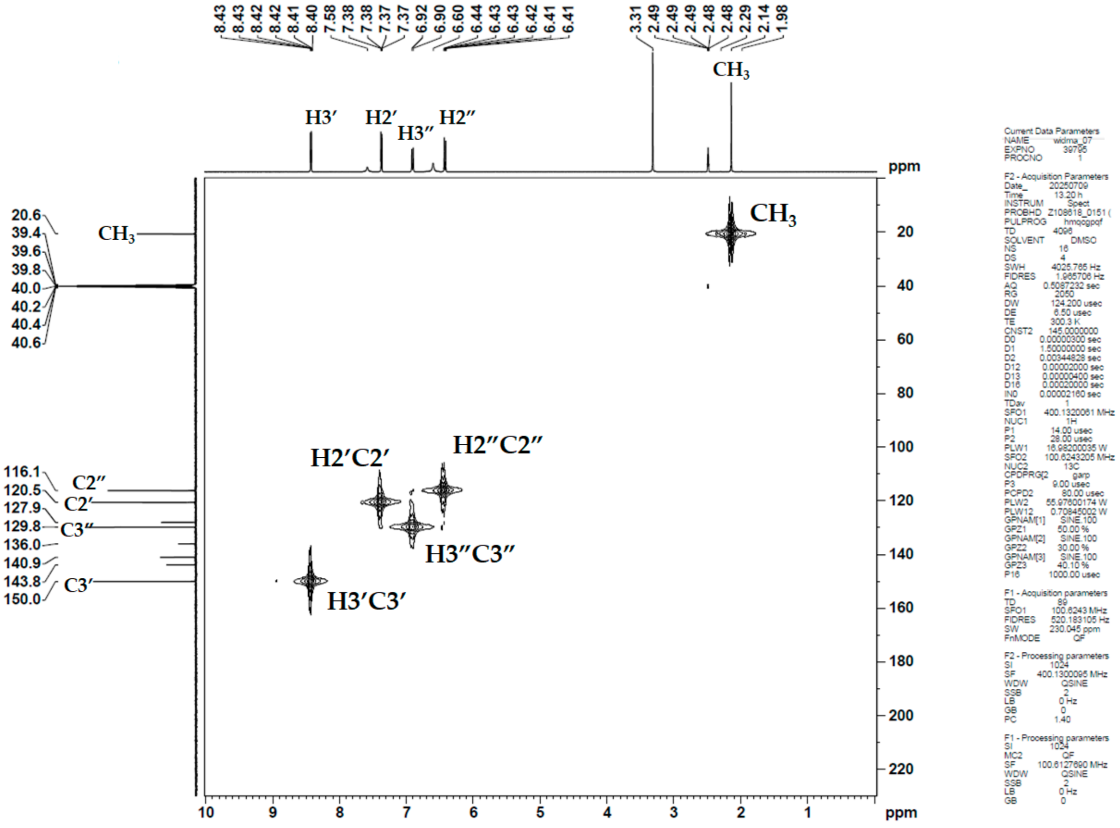

Figure S3. ^{1}H-^{13}C HMQC-NMR spectrum of amidrazone **2g**

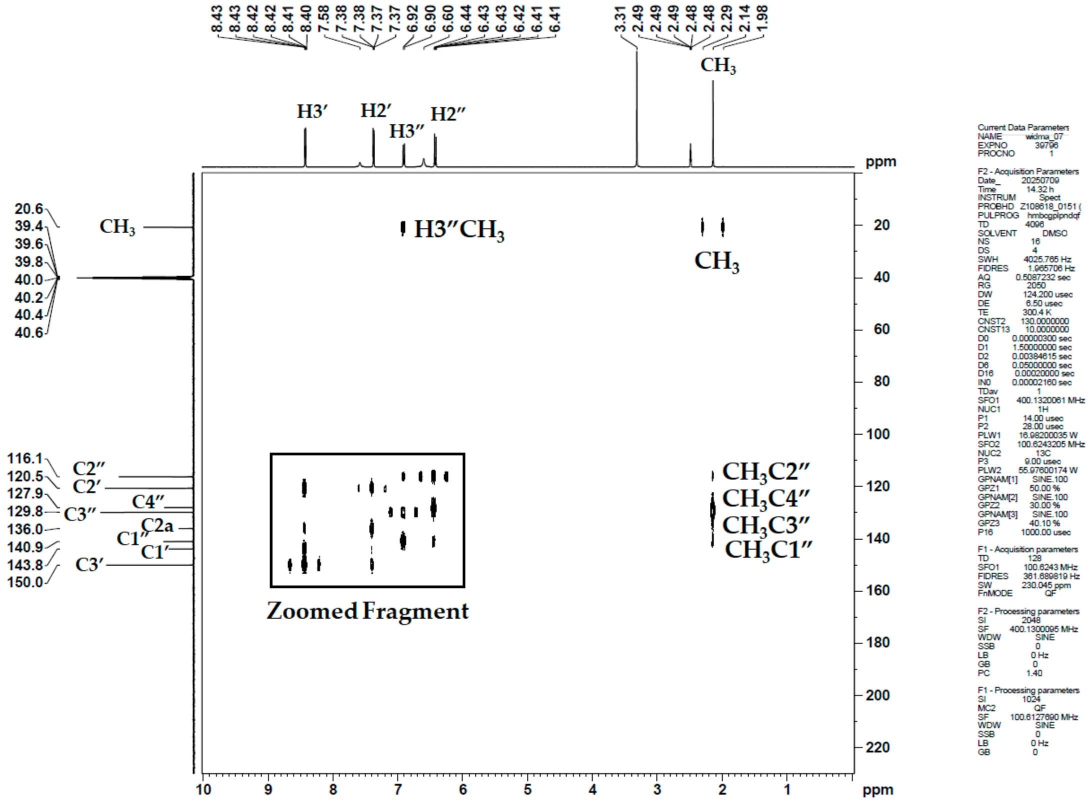

Figure S4. ^{1}H-^{13}C HMBC-NMR spectrum of amidrazone **2g**

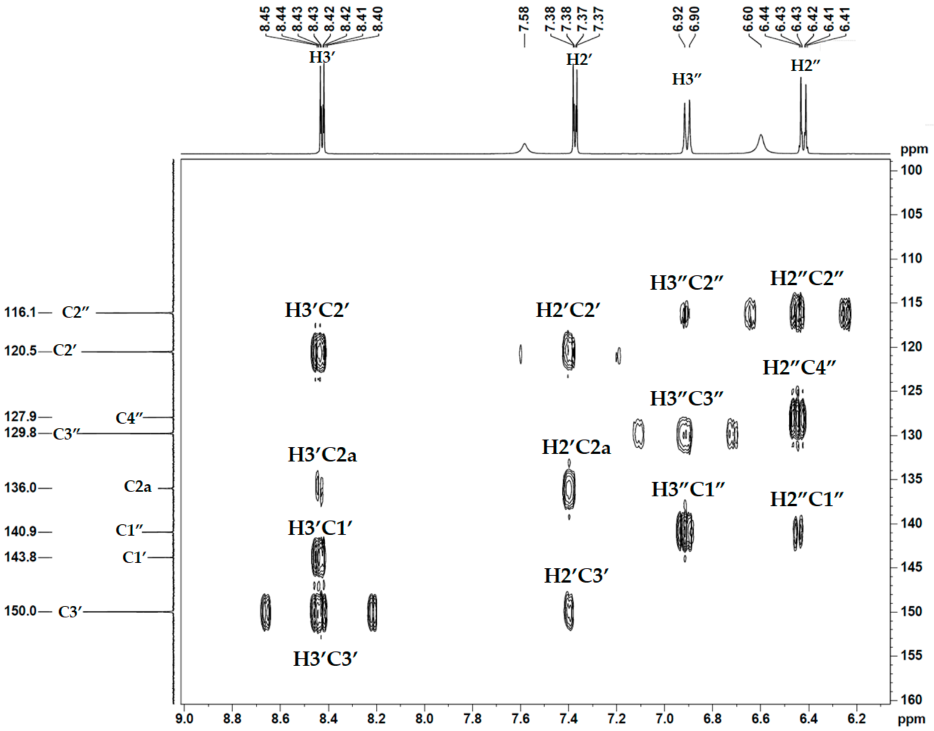

Figure S5. A fragment of the ^{1}H-^{13}C HMBC-NMR spectrum of amidrazone **2g**

PART B. ^{1}H-^{15}N HMBC NMR spectra of amidrazones 2a-2g

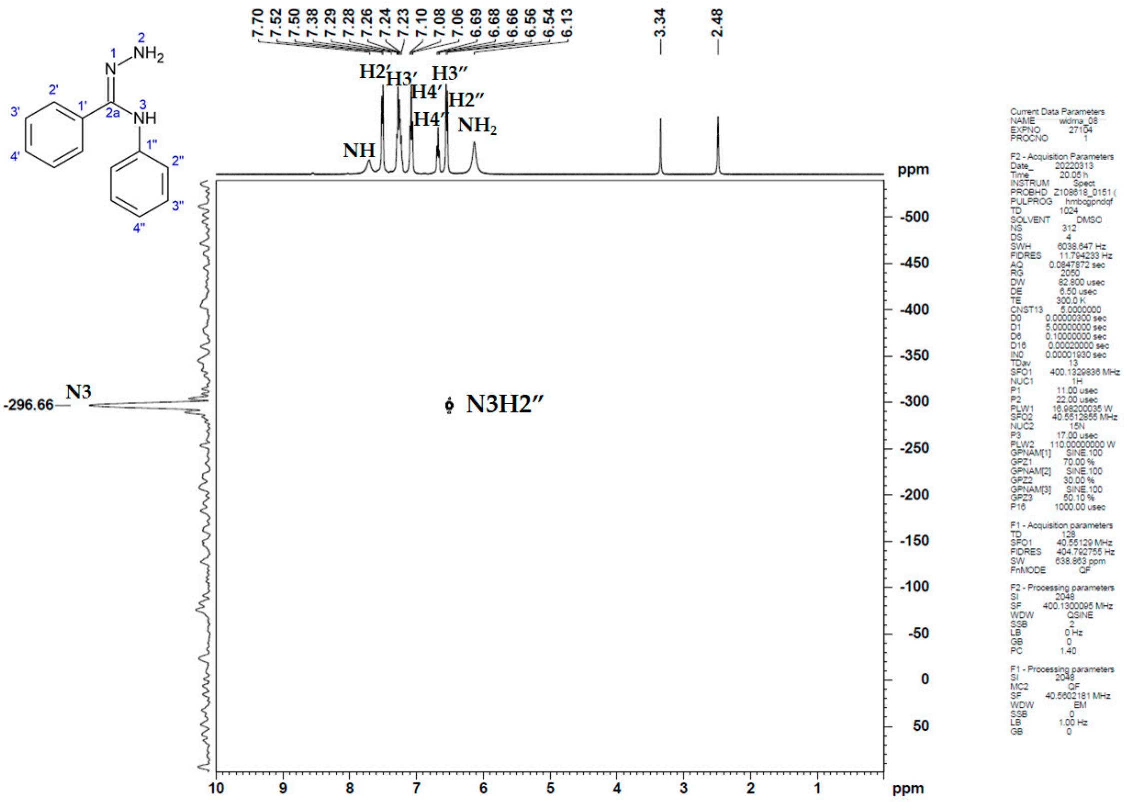

Figure S6. ^{1}H-^{15}N HMBC-NMR spectrum of amidrazone **2a**

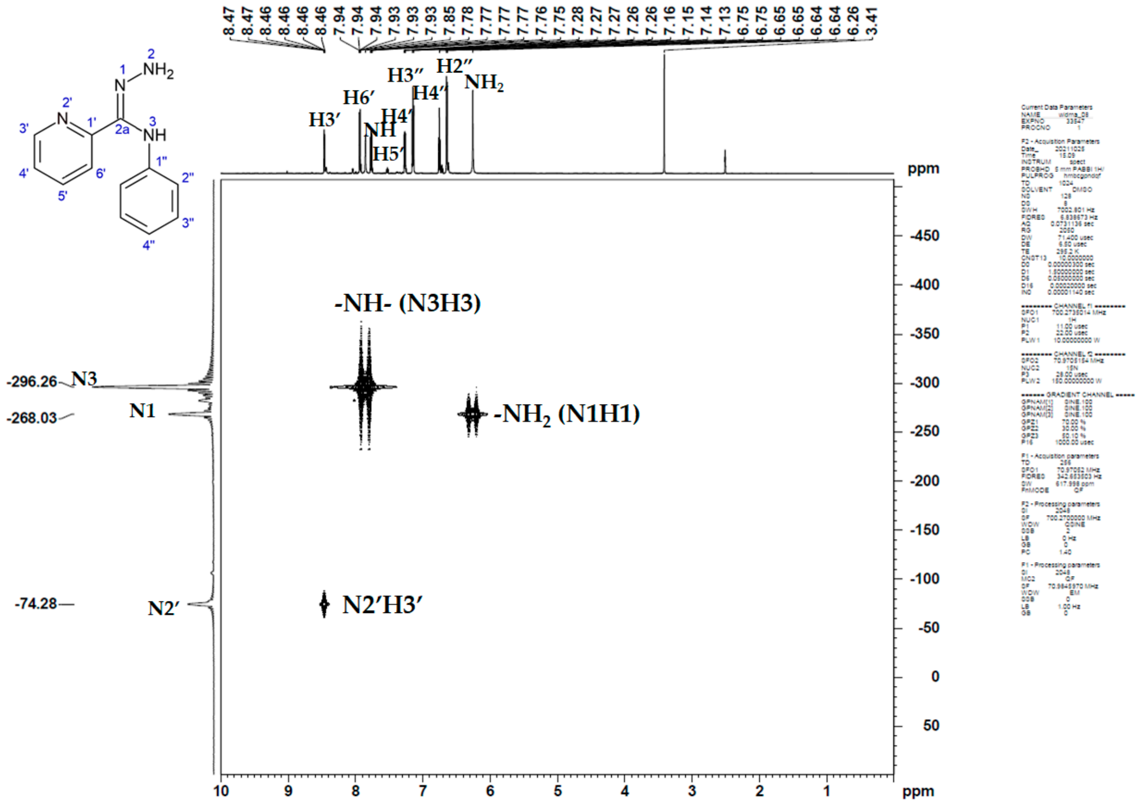

Figure S7. ^{1}H-^{15}N HMBC-NMR spectrum of amidrazone **2b**

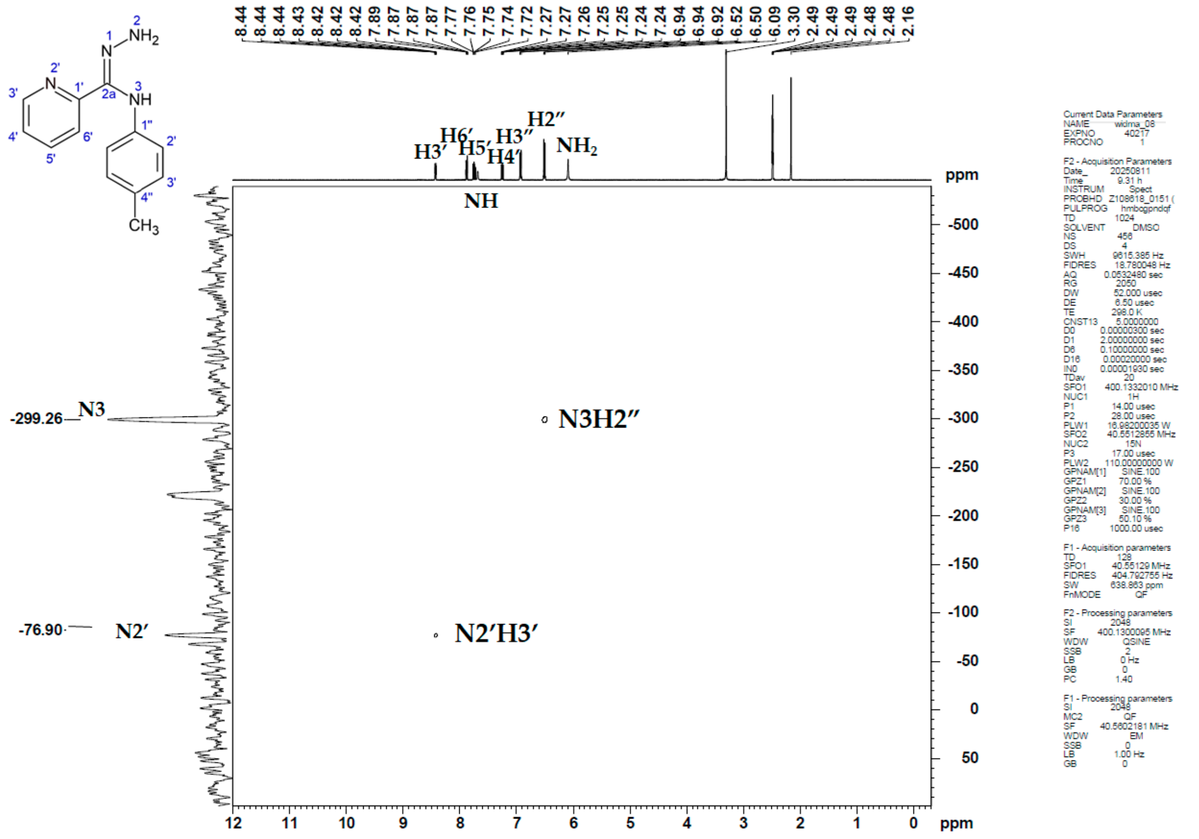

Figure S8. ^{1}H-^{15}N HMBC-NMR spectrum of amidrazone **2c**

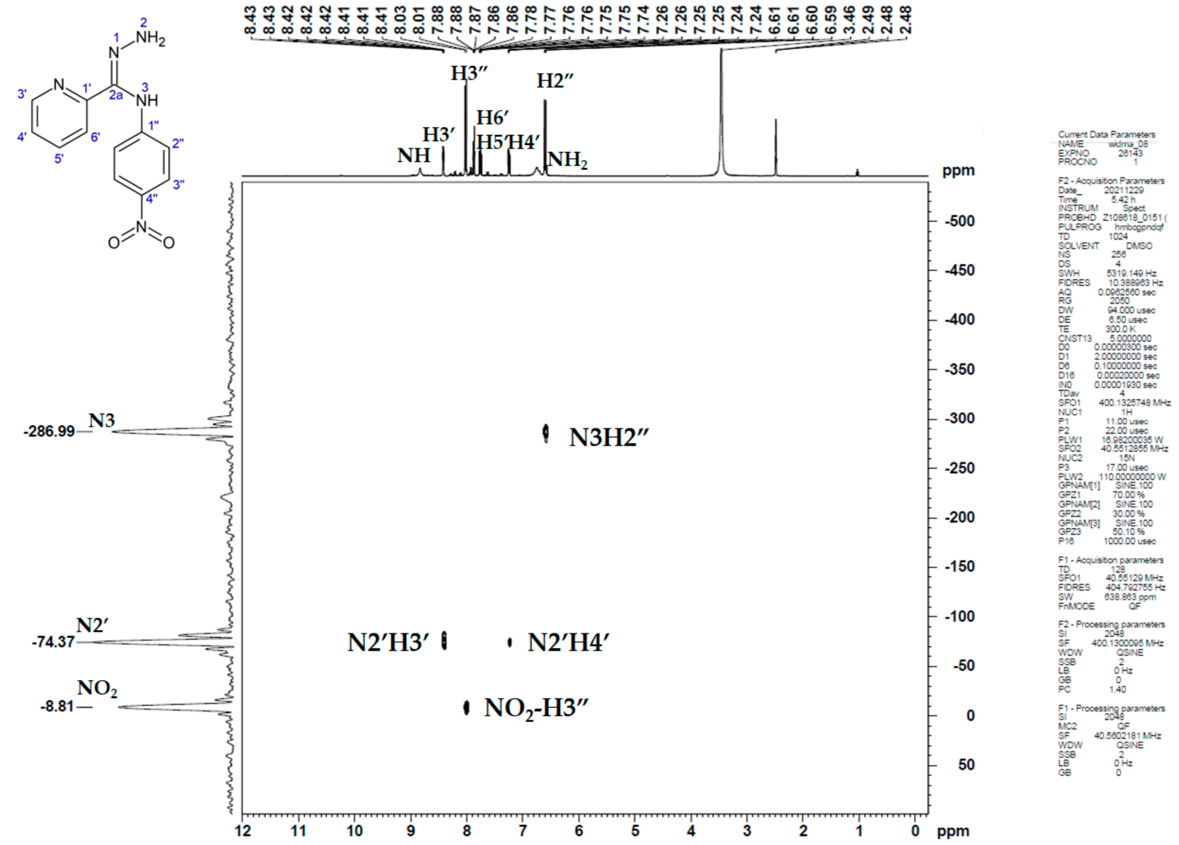

Figure S9. ^{1}H-^{15}N HMBC-NMR spectrum of amidrazone **2d**

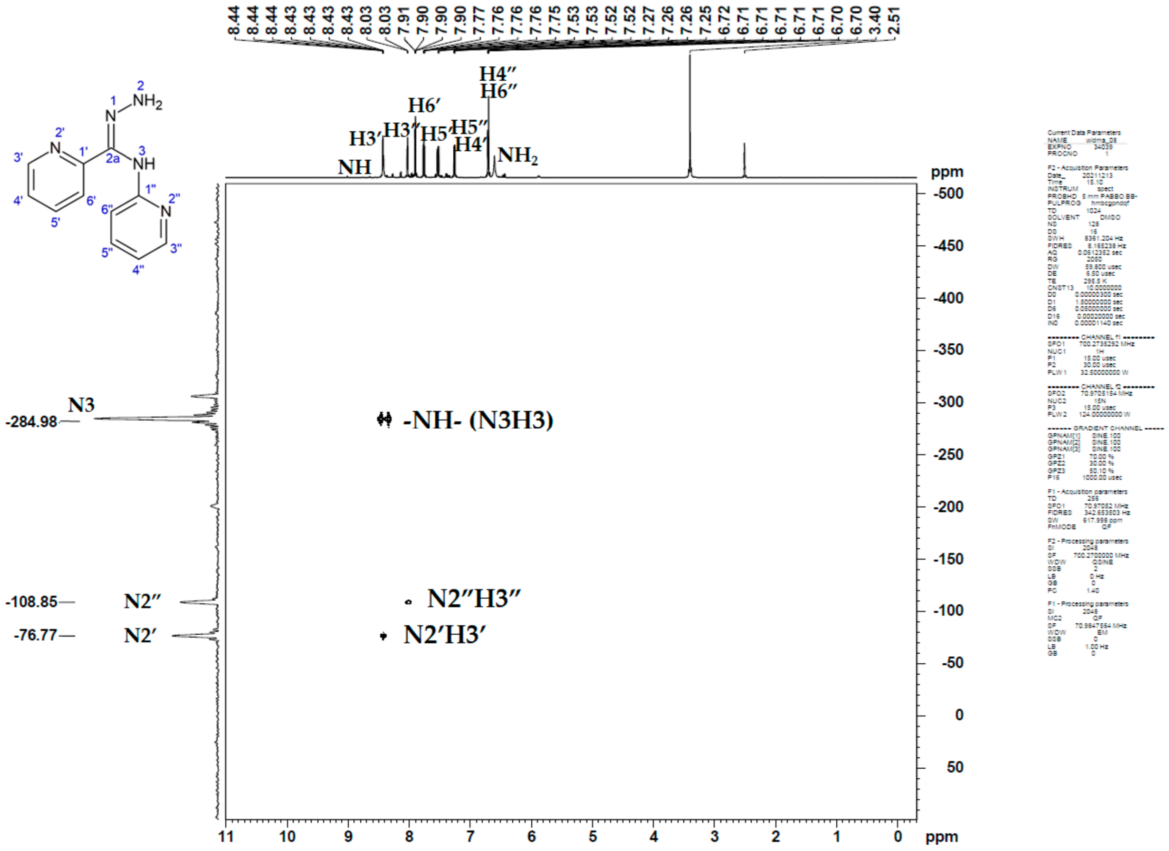

Figure S10. ^{1}H-^{15}N HMBC-NMR spectrum of amidrazone **2e**

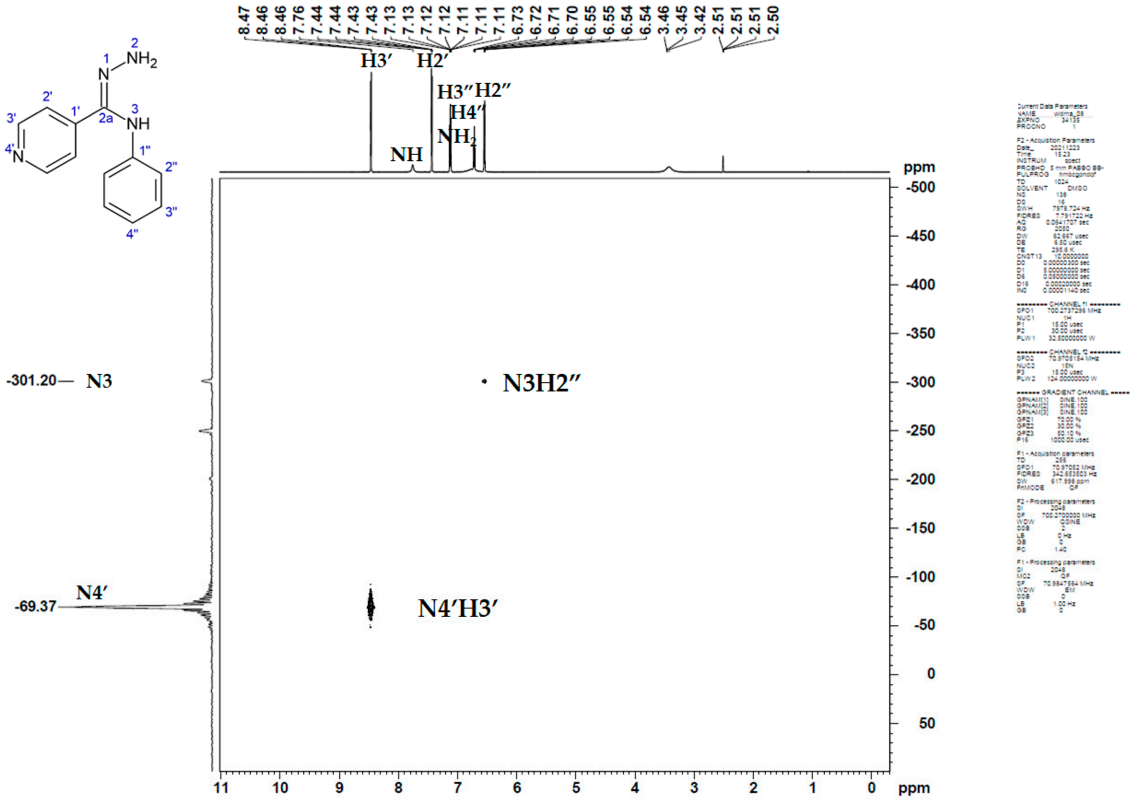

Figure S11. ^{1}H-^{15}N HMBC-NMR spectrum of amidrazone **2f**

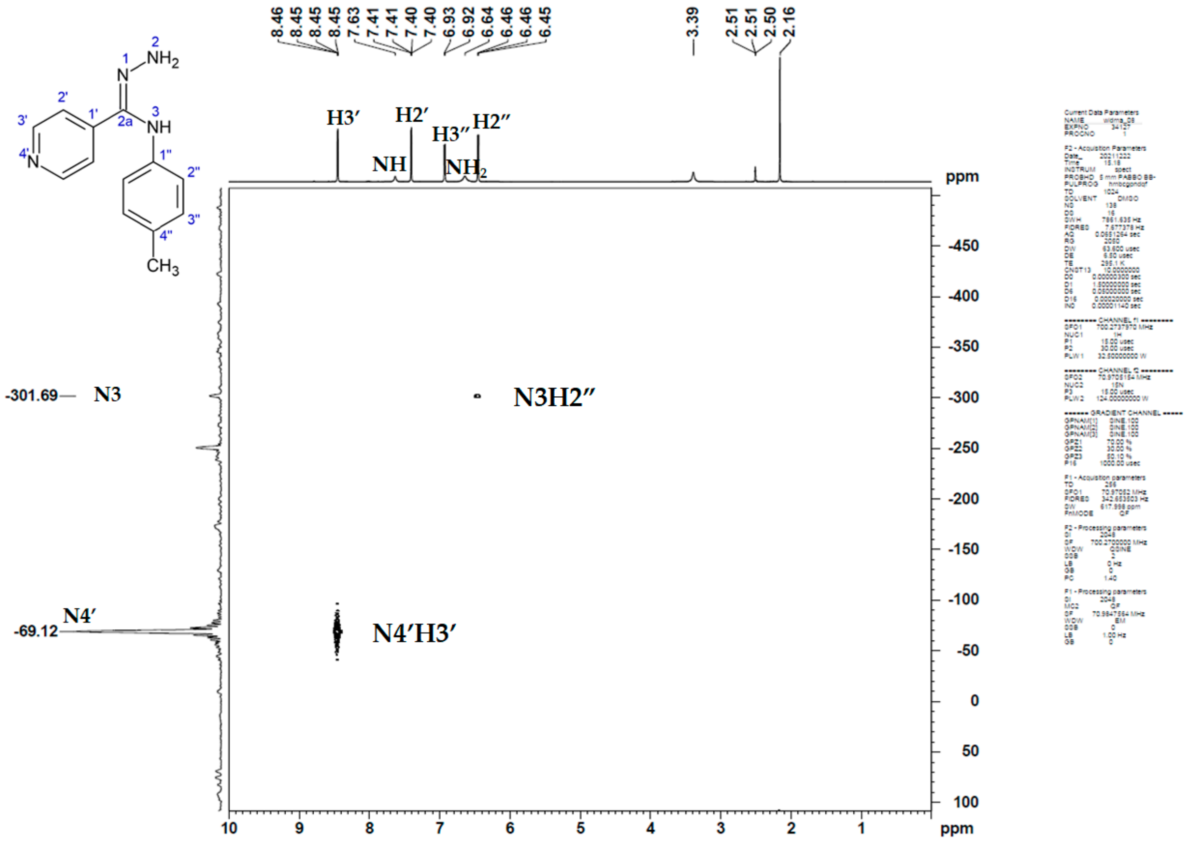

Figure S12. ^{1}H-^{15}N HMBC-NMR spectrum of amidrazone **2g**

PART C. HRMS spectra of amidrazones 2a-2g

Elemental Composition Report

Single Mass Analysis
Tolerance = 5.0 PPM / DBE: min = -1.5, max = 120.0
Element prediction: Off
Number of isotope peaks used for i-FIT = 9

Monoisotopic Mass, Even Electron Ions
24 formula(e) evaluated with 1 results within limits (all results (up to 1000) for each mass)
Elements Used:
C: 0-60 H: 0-50 N: 0-6

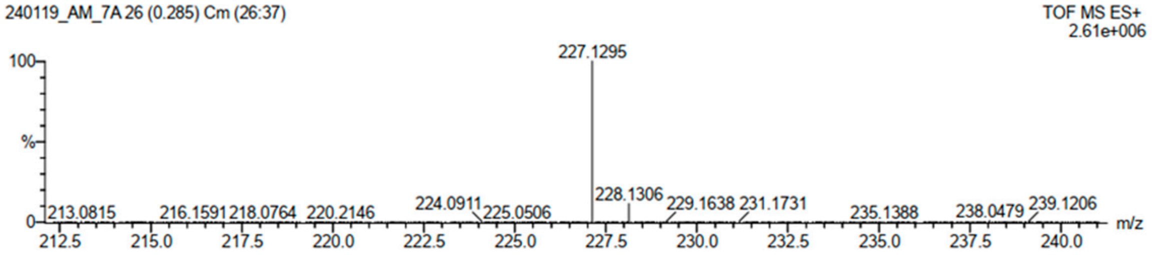

Minimum:				-1.5				
Maximum:		5.0	5.0	120.0				
Mass	Calc. Mass	mDa	PPM	DBE	i-FIT	Norm	Conf(%)	Formula
227.1295	227.1297	-0.2	-0.9	8.5	1794.2	n/a	n/a	C13 H15 N4

Figure S13. HRMS spectrum of amidrazone **2a**

Elemental Composition Report

Single Mass Analysis
Tolerance = 5.0 PPM / DBE: min = -1.5, max = 120.0
Element prediction: Off
Number of isotope peaks used for i-FIT = 9

Monoisotopic Mass, Even Electron Ions
22 formula(e) evaluated with 1 results within limits (all results (up to 1000) for each mass)
Elements Used:
C: 0-60 H: 0-50 N: 0-6

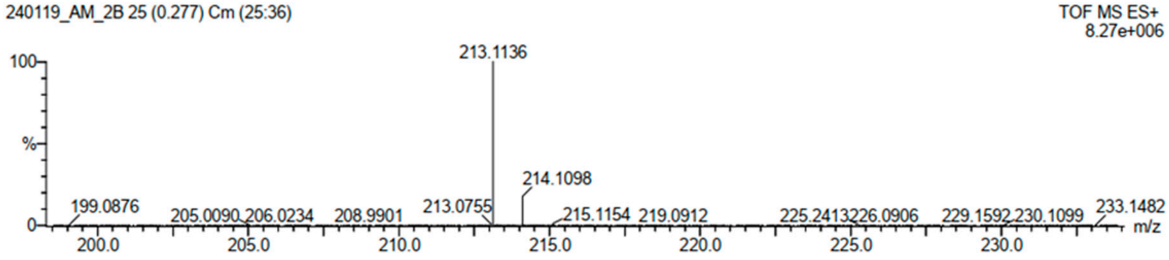

Minimum:				-1.5				
Maximum:		5.0	5.0	120.0				
Mass	Calc. Mass	mDa	PPM	DBE	i-FIT	Norm	Conf(%)	Formula
213.1136	213.1140	-0.4	-1.9	8.5	1958.3	n/a	n/a	C12 H13 N4

Figure S14. HRMS spectrum of amidrazone **2b**

Elemental Composition Report

Single Mass Analysis

Tolerance = 5.0 PPM / DBE: min = -1.5, max = 120.0
Element prediction: Off
Number of isotope peaks used for i-FIT = 9

Monoisotopic Mass, Even Electron Ions
24 formula(e) evaluated with 1 results within limits (all results (up to 1000) for each mass)
Elements Used:
C: 0-60 H: 0-50 N: 0-6

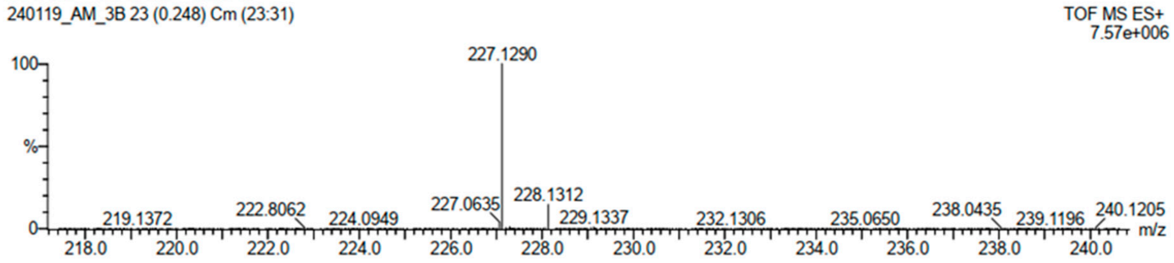

Minimum:				-1.5				
Maximum:		5.0	5.0	120.0				
Mass	Calc. Mass	mDa	PPM	DBE	i-FIT	Norm	Conf(%)	Formula
227.1290	227.1297	-0.7	-3.1	8.5	1995.0	n/a	n/a	C13 H15 N4

Figure S15. HRMS spectrum of amidrazone **2c**

Elemental Composition Report

Single Mass Analysis

Tolerance = 5.0 PPM / DBE: min = -1.5, max = 120.0
Element prediction: Off
Number of isotope peaks used for i-FIT = 9

Monoisotopic Mass, Even Electron Ions
138 formula(e) evaluated with 1 results within limits (all results (up to 1000) for each mass)
Elements Used:
C: 0-60 H: 0-50 N: 0-6 O: 0-5

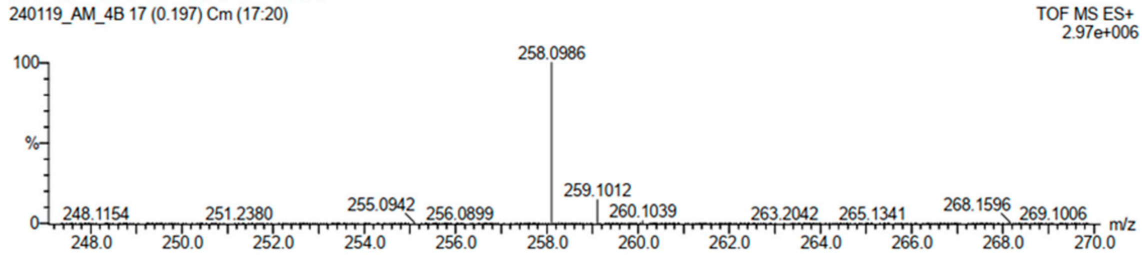

Minimum:				-1.5				
Maximum:		5.0	5.0	120.0				
Mass	Calc. Mass	mDa	PPM	DBE	i-FIT	Norm	Conf(%)	Formula
258.0986	258.0991	-0.5	-1.9	9.5	1500.4	n/a	n/a	C12 H12 N5 O2

Figure S16. HRMS spectrum of amidrazone **2d**

Elemental Composition Report

Single Mass Analysis
Tolerance = 5.0 PPM / DBE: min = -1.5, max = 120.0
Element prediction: Off
Number of isotope peaks used for i-FIT = 9

Monoisotopic Mass, Even Electron Ions
21 formula(e) evaluated with 1 results within limits (all results (up to 1000) for each mass)
Elements Used:
C: 0-60 H: 0-50 N: 0-6
240119_AM_1B 14 (0.160) Cm (13:14)

TOF MS ES+
3.22e+006

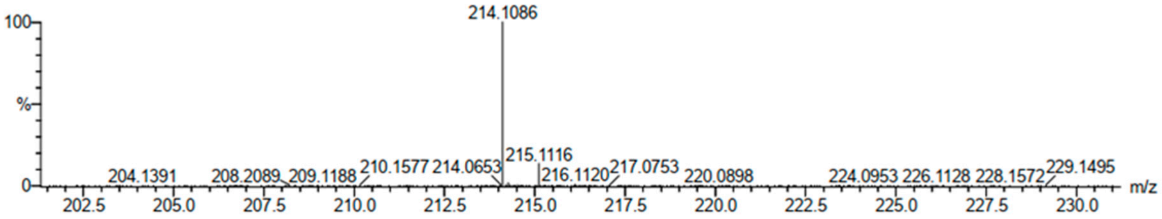

Minimum:				-1.5				
Maximum:		5.0	5.0	120.0				
Mass	Calc. Mass	mDa	PPM	DBE	i-FIT	Norm	Conf(%)	Formula
214.1086	214.1093	-0.7	-3.3	8.5	1150.9	n/a	n/a	C11 H12 N5

Figure S17. HRMS spectrum of amidrazone **2e**

Elemental Composition Report

Single Mass Analysis
Tolerance = 10.0 PPM / DBE: min = -1.5, max = 120.0
Element prediction: Off
Number of isotope peaks used for i-FIT = 9

Monoisotopic Mass, Even Electron Ions
19 formula(e) evaluated with 1 results within limits (all results (up to 1000) for each mass)
Elements Used:
C: 0-60 H: 0-120 N: 0-5
240205_AM6_A 26 (0.285) Cm (26:30-3:8)

TOF MS ES+
6.67e+006

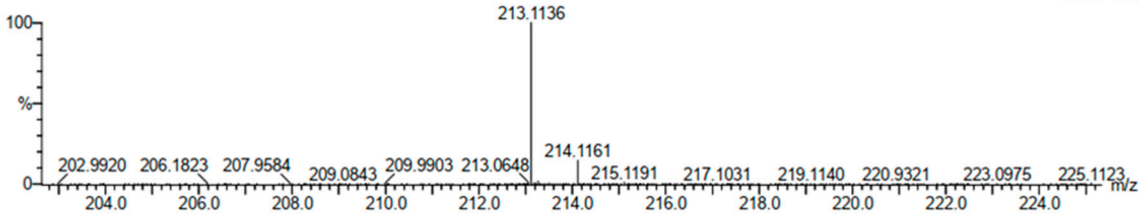

Minimum:				-1.5				
Maximum:		5.0	10.0	120.0				
Mass	Calc. Mass	mDa	PPM	DBE	i-FIT	Norm	Conf(%)	Formula
213.1136	213.1140	-0.4	-1.9	8.5	1393.0	n/a	n/a	C12 H13 N4

Figure S18. HRMS spectrum of amidrazone **2f**

Elemental Composition Report

Single Mass Analysis

Tolerance = 5.0 PPM / DBE: min = -1.5, max = 120.0
Element prediction: Off
Number of isotope peaks used for i-FIT = 9

Monoisotopic Mass, Even Electron Ions
24 formula(e) evaluated with 1 results within limits (all results (up to 1000) for each mass)
Elements Used:
C: 0-60 H: 0-50 N: 0-6

240119_AM_7A 26 (0.285) Cm (26:37)

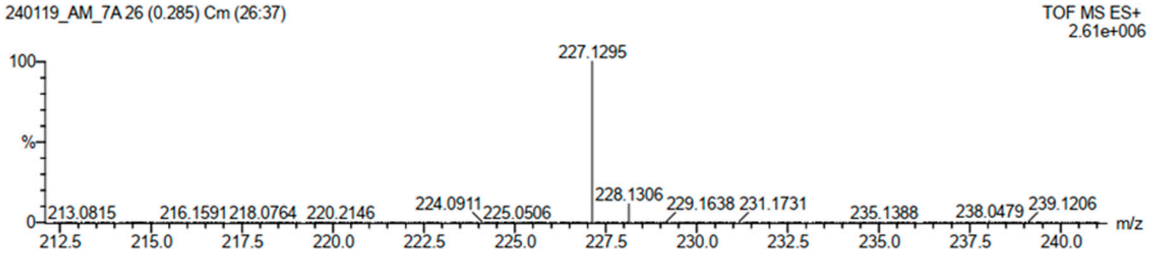

Minimum:				-1.5				
Maximum:		5.0	5.0	120.0				
Mass	Calc. Mass	mDa	PPM	DBE	i-FIT	Norm	Conf(%)	Formula
227.1295	227.1297	-0.2	-0.9	8.5	1794.2	n/a	n/a	C13 H15 N4

Figure S19. HRMS spectrum of amidrazone **2g**
